# Transcriptional responses and flavor volatiles biosynthesis in methyl jasmonate-treated tea leaves

**DOI:** 10.1186/s12870-015-0609-z

**Published:** 2015-09-30

**Authors:** Jiang Shi, ChengYing Ma, DanDan Qi, HaiPeng Lv, Ting Yang, QunHua Peng, ZongMao Chen, Zhi Lin

**Affiliations:** Key Laboratory of Tea Biology and Resource Utilization of Ministry of Agriculture, Tea Research Institute, Chinese Academy of Agricultural Sciences, 9th South Meiling Road, Hangzhou, Zhejiang 310008 PR China; Graduate School of Chinese Academy of Agricultural Sciences, 12 South Street of Zhongguancun, Beijing, 100081 PR China

## Abstract

**Background:**

Tea (*Camellia sinensis*) has long been consumed worldwide for its amazing flavor and aroma. Methyl jasmonate (MeJA), which acts as an effective elicitor among the plant kingdom, could mostly improve the quality of tea aroma by promoting flavor volatiles in tea leaves. Although a variety of volatile secondary metabolites that contribute to aroma quality have been identified, our understanding of the biosynthetic pathways of these compounds has remained largely incomplete. Therefore, information aboaut the transcriptome of tea leaves and, specifically, details of any changes in gene expression in response to MeJA, is required for a better understanding of the biological mechanisms of MeJA-mediated volatiles biosynthesis. Moreover, MeJA treatment could exaggerate the responses of secondary metabolites and some gene expression which offer a better chance to figure out the mechanism.

**Results:**

The results of two-dimensional gas-chromatograph mass-spectrometry showed that the terpenoids content in MeJA-treated tea leaves increased, especially linalool, geraniol, and phenylethyl alcohol. More importantly, we carried out RNA-seq to identify the differentially expressed genes (DEGs) related to volatiles biosynthesis pathways induced by MeJA treatment (0 h, 12 h, 24 h and 48 h) in tea leaves. We identified 19245, 18614, 11890 DEGs respectively in the MeJA_12h, MeJA_24 h and MeJA_48 h samples. The α-Lenolenic acid degradation pathway was firstly responded resulting in activating the JA-pathway inner tea leaves, and the MEP/DOXP pathway significantly exaggerated. Notably, the expression level of jasmonate O-methyltransferase, which is associated with the central JA biosynthesis pathway, was increased by 7.52-fold in MeJA_24 h tea leaves. Moreover, the genes related to the terpenoid backbone biosynthesis pathway showed different expression patterns compared with the untreated leaves. The expression levels of 1-deoxy-D-xylulose-phosphate synthase (DXS), all-trans-nonaprenyl-diphosphate synthase, geranylgeranyl reductase, geranylgeranyl diphosphate synthase (type II), hydroxymethylglutaryl-CoA reductase and 4-hydroxy-3-methylbut-2-enyl diphosphate reductase increased by approximately 2–4-fold.

**Conclusions:**

The results of two-dimension gas-chromatography mass-spectrometry analysis suggested that exogenous application of MeJA could induce the levels of volatile components in tea leaves, especially the geraniol, linalool and its oxides. Moreover, the transcriptome analysis showed increased expression of genes in α-Lenolenic acid degradation pathway which produced massive jasmonic acid and quickly activated holistic JA-pathway inner tea leaves, also the terpenoid backbones biosynthesis pathway was significantly affected after MeJA treatment. In general, MeJA could greatly activate secondary metabolism pathways, especially volatiles. The results will deeply increase our understanding of the volatile metabolites biosynthesis pathways of tea leaves in response to MeJA.

**Electronic supplementary material:**

The online version of this article (doi:10.1186/s12870-015-0609-z) contains supplementary material, which is available to authorized users.

## Background

The tea plant (*Camellia sinensis*) is grown mainly for beverage production. Tea quality is important for its market value and is mostly decided by its taste and aroma. Usually, phenolic compounds are responsible for the color and the taste, while the flavor volatile compounds are fundamental for tea aroma [1–4]. A variety of volatile organic components (VOCs) are present in tea, and although these VOCs are only in minute quantities (i.e., 0.01 % of the total dry weight). They have a significant impact on tea aroma because of their low threshold value and resulting high odor units. Generally, the aroma of brewed tea develops by chemical and biochemical transformations in tea leaves during tea plants’ cultivation, production and processing. And till now, researchers have made progress in determining the main constituents of tea aroma and its formation during manufacturing. The major volatiles in tea leaves are mostly derived from the terpenoid pathways, such as linalool and its oxides, which account for sweet aroma in made tea; or by oxidation of fatty acids, carotenoids, and some amino acid, such as cis-3-Hexenol accounts for the fresh and fruity aroma, and coumarin accounts for the sweet camphoraceous aroma in made tea. All these odor aroma constituents combine to determine the tea aroma quality. According to mass literatures, these progresses are mostly focused on the effects of tea plants’ cultivation, breeding and processing on tea aroma. However, there are limited data on the specific metabolic pathways and molecular mechanisms of the biosynthesis of these odor volatiles [5–8], which hinders progress in determining the underlying mechanisms. Thus, it is important in tea aroma research to identify genes involved in the aroma-related metabolic pathways.

According to the literature, the most convenient and efficient methods to identify the genes related to secondary metabolic pathways are transcriptome combining metabolic analysis after treatment with stress or exogenous elicitors. Plants have the capacity to synthesize, accumulate and emit low-molecular-weight secondary metabolites that are mostly derived from carbohydrate compounds, saturated and unsaturated fatty acids and some amino acids [9, 10]. In particular, when plants experience biotic and abiotic stresses including exogenous elicitors, secondary metabolites biosynthesis pathways are triggered to help plants adapt to the challenging environment [11, 12]. These exogenous stimuli could induce defensive signals directly or indirectly, in addition to subsequent reactions that could extensively change the volatile metabolites profile [13, 14]. To construct an ideal model, it is important to choose an appropriate stimulus. The plant hormone methyl jasmonate acts as an efficient elicitor of secondary metabolite production across the plant kingdom, particularly those involved in a developmental process and defense responses [15, 16]. Several studies have demonstrated that MeJA treatment can trigger the biosynthesis of the volatile secondary metabolites (terpenoids and fatty acid-derived flavor compounds) and non-volatile secondary metabolites (alkaloids, amino acids and phytoalexins) through an extensive transcriptional reprogramming of plant metabolism [17–19]. Besides, MeJA plays an important role in promoting the quality of agricultural products, especially improving the aroma qualities of certain fruits and vegetables, such as apples and strawberries [20–22]. The most important results according to our previous research [23], the tea aroma quality in black tea prepared from MeJA-treated tea leaves was massively promoted. However, the detailed mechanisms of MeJA treatment on tea leaves are still unknown.

There is limited data on the molecular mechanisms of volatiles production in tea compared with other plants. The recently developed deep sequencing technologies represent the most efficient transcript profiling methods available to date. Among these, RNA-seq allows a comparison of the whole transcriptome of tea leaves before and after methyl jasmonate treatment. Comparing the transcriptome before and after MeJA treatment may allow the identification of candidate genes for the biosynthesis of aroma-related metabolites. Hence, we carried out transcriptome using high-throughput Illumina Miseq sequencing and performed volatile metabolite analyses using two-dimensional gas chromatography with time-of-flight mass spectrometry (GC*GC–TOF/MS) to identify the MeJA-responsive volatile secondary metabolic pathways of tea. The identified unigenes were used for subsequent annotation analyses to provide a platform of transcriptome information for genes in tea. In this study, we focused on the identification of terpenoids and certain other volatile metabolism-related genes in tea leaves induced by MeJA. This will provide a basis for further improving tea aroma quality.

## Results and discussions

### Changes in volatile metabolic profile after MeJA treatment

According to the results of two-dimension GC-TOF/MS, the volatile metabolites in MeJA-treated tea leaves changed significantly. We divided the identified metabolites into three groups: C_6_–C_9_, C_10_–C_30_, and the others mainly including some acids. We could clearly figure out that major of content of the flavor volatiles changed significantly after 12 h and 24 h treatment. Most of the volatile compounds in the C_6_–C_9_ category were increased in MeJA_12h treated tea leaves than the ck_12h. 2-Hexenal is important for tea aroma, and responded indirectly to abiotic stress; according to Table 1, the 2-Hexenal content increased to 9.62 μg/g which implied massive biosynthesis of this small-molecular-volatile metabolite after MeJA treatment. Similar results were observed in the C_10_–C_30_ category. Linalool, geraniol, methyl salicylate and phenylethyl alcohol are considered the floral aroma contributors in brewed tea. The content of these four volatile metabolites increased 1.91, 4.4, 0.91 and 9.25 μg/g in MeJA_12h treated tea leaves, respectively, and increased 1.65, 3.58, 5.54 and 5.09 μg/g in MeJA_24 h treated tea leaves, respectively. These results showed a prolonged increase in these four metabolites during MeJA treatment.

**Table 1:** Volatile compounds and some aroma-relative acid precursors in MeJA-treated tea leaves

		Relative content(μg/g)^a^								
Compounds	RT (min)	CK_12h	MJ_12h	T^b^_12h	CK_24h	MJ_24h	T_24h	CK_48h	MJ_48h	T_48h
C6-C9										
2-ethoxy-Butane	5.73	8.42 ± 0.59	10.43 ± 0.66	2.01 ± 0.39	5.69 ± 0.39	17.48 ± 1.72	11.79 ± 1.03	6.42 ± 0.4	10.17 ± 0.84	3.76 ± 0.18
Cyclohexane	6.02	0.95 ± 0.07	1.39 ± 0.09	0.44 ± 0.07	1.05 ± 0.07	4.18 ± 0.41	3.13 ± 0.27	0.62 ± 0.04	1.2 ± 0.1	0.58 ± 0.03
1-ethoxy-Butane^c^	6.11	0 ± 0	1.8 ± 0.11	1.8 ± 0.11	0 ± 0	3.5 ± 0.34	3.5 ± 0.31	0 ± 0	1.21 ± 0.1	1.21 ± 0.06
1-ethoxy-Pentane^c^	7.47	2.98 ± 0.21	4.84 ± 0.3	1.85 ± 0.16	2.4 ± 0.16	6.05 ± 0.59	3.65 ± 0.32	2.52 ± 0.16	2.51 ± 0.21	0 ± 0
Acetic acid, butyl ester	8.73	5.52 ± 0.33	6.11 ± 0.38	0.58 ± 0.24	3.42 ± 0.24	5.9 ± 0.58	2.48 ± 0.22	4.08 ± 0.25	3.62 ± 0.3	−0.47 ± 0.02
2,4-dimethyl-Heptane	9.02	1.61 ± 0.11	3.43 ± 0.22	1.82 ± 0.1	1.45 ± 0.1	2.52 ± 0.25	1.07 ± 0.09	1.59 ± 0.1	1.35 ± 0.11	−0.24 ± 0.01
2-Hexenal^c^	9.93	4.86 ± 0.34	9.62 ± 0.61	4.76 ± 0.21	3.01 ± 0.21	1.79 ± 0.18	−1.22 ± 0.11	1.11 ± 0.07	2.26 ± 0.19	1.15 ± 0.05
4-methyl-Octane^c^	10.27	9.67 ± 0.58	16.18 ± 1.02	6.52 ± 0.84	12.26 ± 0.84	11.06 ± 1.09	−1.2 ± 0.1	11.79 ± 0.73	8.79 ± 0.72	−3 ± 0.14
1-methoxy-3-methyl-Butane	11.07	0.92 ± 0.05	0.98 ± 0.06	0.06 ± 0.04	0.57 ± 0.04	0.94 ± 0.09	0.37 ± 0.03	0.63 ± 0.04	0.6 ± 0.05	−0.03 ± 0
(S)-2-Heptanol^c^	11.47	6.01 ± 0.42	10.54 ± 0.66	4.53 ± 0.62	9.06 ± 0.62	11.78 ± 1.16	2.71 ± 0.24	7.83 ± 0.48	6.82 ± 0.56	−1.01 ± 0.05
1-Octen-3-ol	14.27	1.18 ± 0.08	0.68 ± 0.04	−0.49 ± 0.05	0.7 ± 0.05	0.66 ± 0.06	−0.05 ± 0	0.08 ± 0	0.74 ± 0.06	0.66 ± 0.03
Decane	15.04	1.44 ± 0.1	1.87 ± 0.12	0.43 ± 0.17	2.51 ± 0.17	2.05 ± 0.2	−0.45 ± 0.04	1.64 ± 0.1	2.06 ± 0.17	0.43 ± 0.02
2-ethyl-1-Hexanol	16.06	22.16 ± 1.55	21.82 ± 1.37	−0.34 ± 1.3	18.9 ± 1.3	16.11 ± 1.58	−2.79 ± 0.24	17.51 ± 1.08	25.24 ± 2.07	7.72 ± 0.37
Nonanal	18.93	0.55 ± 0.04	0.58 ± 0.04	0.03 ± 0.03	0.43 ± 0.03	0.59 ± 0.06	0.17 ± 0.01	0.5 ± 0.03	0.4 ± 0.03	−0.1 ± 0
2-methyl-Decane	31.93	1.71 ± 0.12	1.72 ± 0.11	0.01 ± 0.06	0.93 ± 0.06	0.7 ± 0.07	−0.23 ± 0.02	1.18 ± 0.07	0.79 ± 0.06	−0.39 ± 0.02
C10-C30										
(Z)-3-Hexen-1-ol, acetate	15.13	0.85 ± 0.06	1.89 ± 0.12	1.04 ± 0.17	2.47 ± 0.17	2.29 ± 0.23	−0.18 ± 0.02	2.5 ± 0.15	1.77 ± 0.15	−0.73 ± 0.03
Limonene	16.23	0.48 ± 0.02	0.45 ± 0.03	−0.03 ± 0.02	0.3 ± 0.02	0.44 ± 0.04	0.14 ± 0.01	0.33 ± 0.02	0.31 ± 0.03	−0.02 ± 0
Benzyl alcohol	16.41	7.46 ± 0.37	6.9 ± 0.43	−0.56 ± 0.33	4.85 ± 0.33	6.1 ± 0.6	1.25 ± 0.11	5.51 ± 0.34	5.86 ± 0.48	0.34 ± 0.02
3,7-dimethyl-1-Octene	17.86	0.87 ± 0.04	0.82 ± 0.05	−0.04 ± 0.04	0.52 ± 0.04	0.54 ± 0.05	0.02 ± 0	0.6 ± 0.04	0.57 ± 0.05	−0.03 ± 0
Linalool^c^	18.73	6.77 ± 0.43	8.68 ± 0.43	1.91 ± 0.42	6.13 ± 0.42	7.98 ± 0.59	1.65 ± 0.01	6.28 ± 0.39	6.39 ± 0.52	0.11 ± 0.01
Phenylacetaldehyde^c^	18.68	0.89 ± 0.06	1.19 ± 0.07	0.3 ± 0.08	1.24 ± 0.08	0.38 ± 0.04	−0.86 ± 0.07	1.02 ± 0.06	0.56 ± 0.05	−0.45 ± 0.02
Phenylethyl Alcohol^c^	19.33	25.2 ± 1.76	34.45 ± 2.17	9.25 ± 1.88	22.38 ± 1.88	27.49 ± 2.66	5.09 ± 0.03	32.12 ± 1.99	27.33 ± 2.24	−4.79 ± 0.23
Methyl salicylate^c^	22.33	5.78 ± 0.05	6.69 ± 0.47	0.91 ± 0.8	5.6 ± 0.8	11.06 ± 1.09	5.54 ± 0.05	5.14 ± 0.32	12.34 ± 1.01	7.19 ± 0.34
2,3-dihydro-Benzofuran	22.86	5.15 ± 0.46	8.01 ± 0.5	2.87 ± 0.53	7.76 ± 0.53	7.34 ± 0.72	−0.42 ± 0.04	6.97 ± 0.43	8.54 ± 0.7	1.57 ± 0.08
Benzothiazole	23.67	1.38 ± 0.12	0.83 ± 0.05	−0.55 ± 0.03	0.43 ± 0.03	2.3 ± 0.23	1.87 ± 0.16	0.95 ± 0.06	0.47 ± 0.04	−0.48 ± 0.02
Geraniol^c^	24.24	10.48 ± 0.66	13.11 ± 1.18	2.63 ± 0.84	10.23 ± 0.84	14.63 ± 1.44	4.4 ± 0.21	11.05 ± 0.68	10.04 ± 0.82	−1.02 ± 0.05
Indole^c^	25.83	0.93 ± 0.08	4.22 ± 0.27	3.28 ± 0.03	0.49 ± 0.03	2.24 ± 0.22	1.75 ± 0.15	0.64 ± 0.04	0.43 ± 0.04	−0.21 ± 0.01
Coumarin^c^	30.73	1.65 ± 0.12	3.41 ± 0.21	1.76 ± 0.13	1.87 ± 0.13	4.21 ± 0.41	2.34 ± 0.2	1.9 ± 0.12	2.31 ± 0.19	0.41 ± 0.02
Methyl jasmonate^c^	41.68	2.79 ± 0.11	8.21 ± 0.01	5.57 ± 0.12	1.73 ± 0.12	5.63 ± 0.06	3.9 ± 0.1	1.92 ± 0.12	1.91 ± 0.16	−0.01 ± 0
Hexadecanoic acid, butyl ester^c^	49.74	5.64 ± 0.4	6.27 ± 0.4	0.63 ± 0.44	6.46 ± 0.44	23.7 ± 2.33	17.24 ± 1.5	6.15 ± 1.74	6.09 ± 0.5	−0.09 ± 0.06
Heptadecyl acetate	50.21	0.87 ± 0.06	0.4 ± 0.02	−0.48 ± 0.04	0.54 ± 0.04	1.56 ± 0.15	1.02 ± 0.09	1.92 ± 0.12	0.57 ± 0.05	−1.35 ± 0.06
(Z)-9-Octadecenamide	53.32	2.41 ± 0.17	2.07 ± 0.13	−0.34 ± 0.25	3.61 ± 0.25	1.75 ± 0.17	−1.86 ± 0.16	3.31 ± 0.2	2.52 ± 0.21	−0.79 ± 0.04
Octadecanoic acid, 2-methylpropyl ester	53.86	3.37 ± 0.24	3.21 ± 0.2	−0.15 ± 0.27	3.96 ± 0.27	13.2 ± 1.3	9.25 ± 0.81	3.62 ± 1.09	3.33 ± 0.27	−0.28 ± 0.08
Dodecanamide	53.87	1.26 ± 0.15	0.58 ± 0.04	−0.67 ± 0.05	0.78 ± 0.05	0.48 ± 0.05	−0.3 ± 0.03	0.85 ± 0.05	0.56 ± 0.05	−0.29 ± 0.01
Others										
Salicylic acid^c^	25.46	8.09 ± 0.73	2.39 ± 0.15	−5.7 ± 0.53	7.74 ± 0.53	7.03 ± 0.69	−0.71 ± 0.06	5.07 ± 0.31	8.71 ± 0.72	3.64 ± 0.17
Geranic acid	27.41	0.82 ± 0.07	0.61 ± 0.04	−0.21 ± 0.07	1.03 ± 0.07	1.78 ± 0.18	0.75 ± 0.07	0.98 ± 0.06	0.92 ± 0.08	−0.06 ± 0
Jasmonic acid^c^	15.46	3.49 ± 0.24	29.75 ± 0.15	26.26 ± 0.15	3.16 ± 0.15	16.5 ± 0.44	13.34 ± 0.2	2.4 ± 0.15	2.18 ± 0.18	−0.23 ± 0.01
trans-Cinnamic acid^c^	29.87	1.38 ± 0.1	1.15 ± 0.07	−0.23 ± 0.11	1.56 ± 0.11	3.39 ± 0.33	1.83 ± 0.16	1.62 ± 0.1	1.96 ± 0.16	0.34 ± 0.02
4-hydroxy-Benzoic acid^c^	32.13	2.12 ± 0.15	0.53 ± 0.03	−1.59 ± 0.13	1.95 ± 0.13	1.56 ± 0.15	−0.38 ± 0.03	2.67 ± 0.17	2.23 ± 0.18	−0.45 ± 0.02
Homovanillic acid^c^	36.62	1.33 ± 0.09	2.16 ± 0.14	0.84 ± 0.08	1.21 ± 0.08	8.44 ± 0.83	7.23 ± 0.63	1.45 ± 0.09	0.97 ± 0.08	−0.47 ± 0.02
trans p-Coumaric acid^c^	38.41	1.27 ± 0.09	0.4 ± 0.03	−0.86 ± 0.08	1.18 ± 0.08	1.19 ± 0.12	0 ± 0	0.87 ± 0.05	0.9 ± 0.07	0.02 ± 0
n-Hexadecanoic acid	44.53	4.67 ± 0.19	3.35 ± 0.21	−1.32 ± 0.25	3.59 ± 0.25	3.26 ± 0.32	−0.33 ± 0.03	5.73 ± 0.35	4.09 ± 0.34	−1.63 ± 0.08
(Z,Z)-9,12-Octadecadienoic acid	48.47	1.62 ± 0.06	1.1 ± 0.07	−0.52 ± 0.08	1.1 ± 0.08	1.74 ± 0.17	0.64 ± 0.06	1.76 ± 0.23	1.38 ± 0.11	−0.38 ± 0.01
(Z,Z,Z)-9,12,15-Octadecatrienoic acid^c^	48.63	1.22 ± 0.05	0.03 ± 0	−1.19 ± 0.05	0.75 ± 0.05	0.09 ± 0.01	−0.67 ± 0.06	0.84 ± 0.05	0.98 ± 0.08	0.14 ± 0.01
Octadecanoic acid^c^	49.13	2.24 ± 0.09	1.06 ± 0.07	−1.18 ± 0.09	2.31 ± 0.09	1.53 ± 0.15	−1.22 ± 0.02	2.4 ± 0.15	1.34 ± 0.11	−1.06 ± 0.05

^a^ Relative content (μg/g) represents volatile compounds content in every gram of fresh tea leaves. ^b^ represents content of the compounds in MeJA_12h minus content of the compounds in CK_12h, also means the differences between MeJA_12h and CK_12h; represents content of the compounds in MeJA_24h minus content of the compounds in CK_24h, also means the differences between MeJA_24h and CK_24h; represents content of the compounds in MeJA_48h minus content of the compounds in CK_48h, also means the differences between MeJA_48h and CK_48h. ^c^represents the most important and affected volatile compounds after methyl jasmonate treatment

Much more interestingly, we found most of the acid precursors, such as Salicylic acid, trans-Cinnamic acid, Homovanillic acid, trans p-Coumaric acid, the majority of which are related to volatile biosynthesis pathways, decreased in MeJA-treated 24 h tea leaves. We hypothesized that these acids were consumed as precursors in synthesis volatiles faster than they could be biosynthesized, resulting increased volatiles contents and decreased contents of acid precursors.

Also the content of Octadecanoic acid was decreased significantly after methyl jasmonate treatment, it is the intermediates of the α-linolenic acid metabolism pathway which finally synthesis massive JA and methyl jasmonate (Table 1).

### Illumina sequencing and data analysis

RNA sequencing of the eight samples produced more than 27 million 100 bp paired-end reads, with an average of 3 million reads for each sample. Cleaning and quality checks were carried out on the raw data. More than 18 million trimmed reads were obtained with useful data percentage ranging from 58.96 % to 72.23 %, and the average length of each read was 195 bp (Additional file 1: Table S1). Compared with the reads generated by the formal platforms, the longer length of Illumina Miseq sequencing reads aided the accuracy of the subsequent de novo assembly, despite the lack of an available reference genome for tea. The de novo assembly was performed using Trinity (http://trinityrnaseq.github.io/). All the short reads were assembled to generate 625,574 contigs with a mean size of 290.15 bp and an N50 of 382 bp; 11.13 % reads of the samples were greater than 500 bp. Further assembly of the contigs generated components that were used to construct a de Bruijn graph. Finally, optimizing the de Bruijn graph permitted us to build 320573 transcripts with average size of 796 bp and an N50 of 1392 bp (Table 2). All the transcripts were then BLAST searched against *Arabidopsis* database. For those sequences with no BLAST hits (non-BLASTable transcripts), we searched them against the NCBI non-redundant (nr) database, using the BLASTx program with an E-value threshold of 1E-5. To distinguish redundant sequences from homologous sequences, unigenes were used in this study to minimize redundancy: each unique sequence was assigned a unigene ID according to the accession number of the best-hit homolog in the nr database. 50732 unigenes were obtained, with an average length of 1151 bp (Table 2). The size distribution of contigs, transcripts and unigenes was compiled (Additional file 2: Figure S1).

**Table 2:** Statistical summary of cDNA sequences of tea generated by Illumina Miseq platform

	Total Length(bp)	Sequence NO.	Max Length(bp)	Average Length(bp)	N50	>N50 Reads NO.
Contigs	181,510,070	625,574	35,349	290.15	382	103,855
Transcripts	255,154,143	320,573	19,361	796	1392	52,840
Unigenes	58,385,017	50,723	19,361	1151	1810	10,394

EggNOG (evolutionary genealogy of genes: Non-supervised Orthologous Groups) is a database providing orthologous groups for 943 bacteria, 69 archaea and 121 eukaryotes. According to previous studies, the proteins could be divided into 25 functional categories [24]. Out of 45745 unigenes with significant identity with nr database in this study, 40245 could be classified into 26 eggNOG categories (Additional file 3: Figure S2). The categories “function unknown” (8513, 21.15 %) and “general function prediction only” (7383, 18.35 %) were the two largest functional groups among the eggNOG categories. The high percentage of unigenes classified into “general function prediction only” was similar to transcriptome studies of other species [25–28]. The assignment of so many differential expressed unigenes to the unknown function group suggested the presence of as yet unknown mechanisms of secondary metabolism changes during the MeJA treatment of tea leaves. The next most abundant groups were “Signal transduction mechanisms” (3330, 8.27 %), “Posttranslational modification”, “protein turnover”, “chaperones” (3259, 8.10 %), “Translation”, “ribosomal structure and biogenesis” (1964, 4.88 %), “Transcription” (1847, 4.59 %), whereas the groups involving “cell motility” and “extracellular structures” consisted of a total of 80 unigenes (0.2 %), representing the smallest eggNOG classifications, excepting for two undetermined unigenes. Notably, 1734 unigenes (4.31 %) and 1312 unigenes (3.05 %) were classified into the carbohydrate metabolism and secondary metabolite biosynthesis groups, respectively, including volatile compounds biosynthesis.

### Differentially expressed gene analysis

**Fig. 1:**
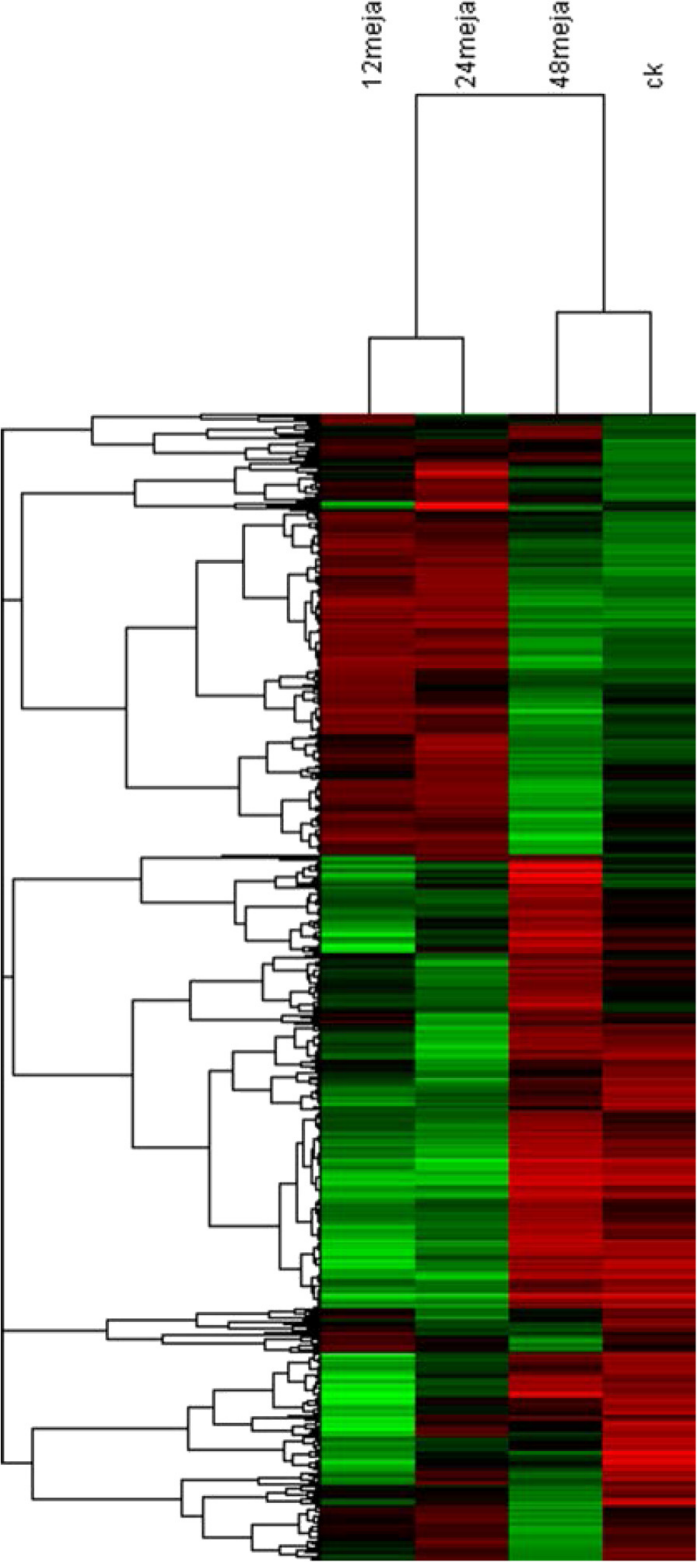
Cluster of differentially expressed unigenes during MeJA treatment. Expression changes and cluster analysis of 10,765 genes that were differentially expressed between any two of four samples. Each row represents a differentially expressed gene, while each column represents a sample. Changes in expression levels are shown in color scales with saturation at >2.0-fold changes. Green and red color gradients indicate a decrease and increase in transcript abundance, respectively

To identify DEGs among MeJA-treated tea samples, we compared them with each other and identified unigenes that were at least 2-fold up- or down regulated between the two samples, with *p*-value less than 0.05. Then, hierarchical clustering was used to gain a global view of DEGs (Fig. 3). The DEGs analysis of the MeJA_12h treated samples was similar to the MeJA_24h treated samples. In total, 19245, 18614DEGs were identified in the MeJA_12h, MeJA_24h samples, respectively (Fig. 1). These two are much more different from the MeJA-untreated ones. Thus, it was clear that MeJA has a significant impact on the transcriptome of tea leaves. However, it also could be deduced from the heat map that the MeJA_48 h samples were much more special. It was different from the others, comparing with others, 11890 DEGs were identified in the MeJA_48 h samples (Fig. 1), and the GO categories for the up- and down-regulated DEGs are shown separately for the three main terms.

It was supposed that various genes were greatly affected within 48 h by MeJA treatment. However, most of DEGs in 12, 24 h-MeJA samples are absolutely not the same as in 48 h-MeJA and CK samples. Mostly, the expression of DEGs was improved within 24 h, then down-regulated. We also know about that the MeJA treatment was much similar to herbivorous attack that finally leading to mass consumption of plant its own. Expression of Genes, proteins and content of metabolomics were firstly improved, then be consumed, and to the last, recovered to the normal level.

**Fig. 2:**
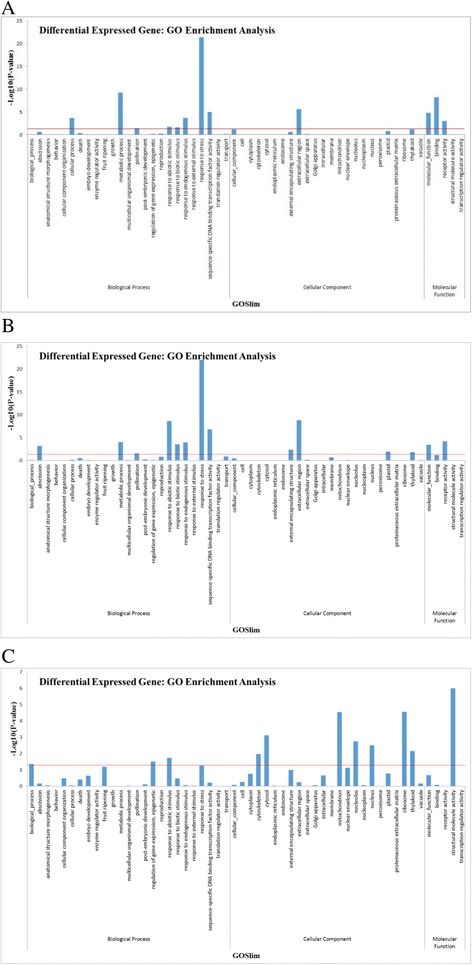
Gene Ontology enrichment assigned to tea unigenes. GO categories of biological process, cellular component and molecular function for the transcriptome of AR. Histogram presentation of the gene ontology classification. The results are summarized in the three main GO categories: biological process, cellular component and molecular function. **a**. Gene Ontology classification of 12 h methyl jasmonate-induced tea leaves; **b**. Gene Ontology classification of 24 h methyl jasmonate-induced tea leaves; **c**. Gene Ontology classification of 48 h methyl jasmonate-induced tea leaves. Note: red line represents the *p* value = 0.05

The KEGG (Kyoto Encyclopedia of Genes and Genomes) is a database linking genomic information with higher order functional information by collecting manually drawn pathway maps representing current knowledge on cellular processes and standardized gene annotations. To gain an overview of tea metabolic pathways that are modulated by MeJA, DEGs were analyzed according to the Kyoto Encyclopedia of Genes and Genomes (KEGG; http://www.genome.jp). The analysis revealed a total of 45 KEGG pathways containing 20404 assigned unigenes (Additional file 4: Table S2). The pathways with the largest numbers of assigned unigenes were “metabolism”, “human disease” and “genetic information processing”. Furthermore, we performed KEGG enrichment analysis of the DEGs identified in the MeJA_12h, MeJA_24h and MeJA_48h samples compared with the MeJA-untreated samples respectively, and picked out 1406, 1443, 1695 DEGs which important in volatile related pathways (Fig. 2).

A list of secondary metabolic pathways represented by the unigenes is provided in Table 3. Interestingly, among the secondary metabolic processes, terpenoids and phenylpropanoid pathways were the most represented (Additional file 4: Table S2; Table 1). Strangely, these volatile secondary metabolite types were rarely reported to accumulate at high levels in tea leaves after MeJA treatment; obviously, the DEGs involved in the biosynthesis of these metabolites were not clearly identified.

**Table 3:** KEGG pathway analysis of the MeJA-responsive differential expressed unigenes

Pathway	Enzyme	Seq	Enzyme ID	Pathway ID	Fold changes of DE unigenes		
Fatty acid relative metabolism							
alpha-Linolenic acid metabolism					MeJA_12h	MeJA_24h	MeJA_48h
	allene oxide cyclase	comp48042_c0_seq1	EC:5.3.99.6	map00592	−2.5	−2.33	
	acyl-CoA oxidase	comp107278_c0_seq1	EC:1.3.3.6	map00592	2.37	4.24	1.11
	LOX2S; lipoxygenase	comp120346_c1_seq33	EC:1.13.11.12	map00592	6.81	5.1	
	LOX3S; lipoxygenase	comp120346_c1_seq24	EC:1.13.11.12	map00592		5.1	
	jasmonate O-methyltransferase	comp108303_c0_seq1	EC:2.1.1.141	map00592	3.97	7.52	
	ACAA1; acetyl-CoA acyltransferase 1	comp97676_c0_seq1	EC:2.3.1.16	map00592		−2.13	
Linoleic acid metabolism							
	LOX1_5; linoleate 9S-lipoxygenase	comp115097_c0_seq1	EC:1.13.11.58	map00591	−2.13		
Biosynthesis of unsaturated fatty acids							
	DESA1; acyl-[acyl-carrier-protein] desaturase	comp108284_c0_seq2	EC:1.14.19.2	map01040	−47.62		
	FAD2; omega-6 fatty acid desaturase (delta-12 desaturase)	comp103729_c0_seq1	EC:1.14.19.-	map01040	−4		
	SCD; stearoyl-CoA desaturase (delta-9 desaturase)	comp108284_c0_seq2	EC:1.14.19.1	map01040	−47.62	−66.67	−76.92
	FAD8; omega-3 fatty acid desaturase (delta-15 desaturase)	comp118154_c5_seq3	EC:1.14.19.-	map01040	2.73	3.78	2.63
	HADHA; enoyl-CoA hydratase / long-chain 3-hydroxyacyl-CoA dehydrogenase	comp110054_c0_seq1	EC:4.2.1.17 1.1.1.211	map01040			18.24
	PHS1; very-long-chain (3R)-3-hydroxyacyl-[acyl-carrier protein] dehydratase	comp26306_c0_seq1	EC:4.2.1.134	map01040			31.15
Amino acid relative metabolism							
Arginine and proline metabolism							
	pyrroline-5-carboxylate reductase	comp109262_c0_seq1	EC:1.5.1.2	map00330	−2.33	−3.23	
	creatine kinase	comp115225_c0_seq1	EC:2.7.3.2	map00330	−2.13	−4.17	40.19
	aldehyde dehydrogenase (NAD+)	comp101398_c0_seq1	EC:1.2.1.3	map00330		2.26	
	PRODH; proline dehydrogenase	comp106217_c0_seq1	EC:1.5.-.-	map00330		4.57	1.18
	spermidine synthase	comp107047_c0_seq3	EC:2.5.1.16	map00330		12.78	
	arginase	comp114916_c0_seq1	EC:3.5.3.1	map00330		3.41	3.14
	speD; S-adenosylmethionine decarboxylase	comp107569_c0_seq9	EC:4.1.1.50	map00330		4.28	
	glnA; glutamine synthetase	comp100912_c0_seq1	EC:6.3.1.2	map00330		3.12	1.05
	ALDH18A1; delta-1-pyrroline-5-carboxylate synthetase	comp107259_c0_seq7	EC:2.7.2.11 1.2.1.41	map00330		2.98	
	argAB; amino-acid N-acetyltransferase	comp121994_c0_seq4	EC:2.3.1.1	map00330		2.18	
	ornithine decarboxylase	comp114034_c0_seq1	EC:4.1.1.17	map00330		−3.13	−1.12
	GLUD1_2; glutamate dehydrogenase (NAD(P)+)	comp117818_c0_seq2	EC:1.4.1.3	map00330			45.52
	CNDP2; cytosolic nonspecific dipeptidase	comp91028_c0_seq1	EC:3.4.13.18	map00330			14.85
Valine, leucine and isoleucine biosynthesis							
	ilvC; ketol-acid reductoisomerase	comp97612_c1_seq1	EC:1.1.1.86	map00290	−20.41	−19.23	
	branched-chain amino acid aminotransferase	comp119162_c0_seq8	EC:2.6.1.42	map00290	71.4	65.24	
	leuA; 2-isopropylmalate synthase	comp121716_c0_seq8	EC:2.3.3.13	map00290		2.14	
Valine, leucine and isoleucine degradation							
	aldehyde dehydrogenase (NAD+)	comp101398_c0_seq1	EC:1.2.1.3	map00280	2.44	2.26	
	HIBCH; 3-hydroxyisobutyryl-CoA hydrolase	comp117162_c1_seq5	EC:3.1.2.4	map00280		2.44	2.17
	ACADM; acyl-CoA dehydrogenase	comp120137_c0_seq1	EC:1.3.8.7	map00280			22.02
	DLD; dihydrolipoamide dehydrogenase	comp104094_c0_seq1	EC:1.8.1.4	map00280			31.87
	ACAA2; acetyl-CoA acyltransferase 2	comp80532_c0_seq1	EC:2.3.1.16	map00280			18.16
	HADHB; acetyl-CoA acyltransferase	comp88765_c0_seq1	EC:2.3.1.16	map00280			9.36
	ECHS1; enoyl-CoA hydratase	comp110838_c0_seq1	EC:4.2.1.17	map00280			29.37
	HADHA; enoyl-CoA hydratase / long-chain 3-hydroxyacyl-CoA dehydrogenase	comp110054_c0_seq1	EC:4.2.1.17 1.1.1.211	map00280			18.24
	ACADSB; short/branched chain acyl-CoA dehydrogenase	comp102774_c0_seq1	EC:1.3.99.12	map00280			67.47
Phenylalanine metabolism							
	TAT; tyrosine aminotransferase	comp119529_c0_seq30	EC:2.6.1.5	map00400	−3.85		
	peroxidase	comp119870_c0_seq14	EC:1.11.1.7	map00400	−2		1.03
	trpB; tryptophan synthase beta chain	comp123596_c0_seq1	EC:4.2.1.20	map00400	7.84		
	aroB; 3-dehydroquinate synthase	comp113795_c0_seq1	EC:4.2.3.4	map00400	2.01		
	chorismate mutase	comp112016_c0_seq2	EC:5.4.99.5	map00400	3.06		
	ADT; arogenate/prephenate dehydratase	comp122512_c1_seq4	EC:4.2.1.91 4.2.1.51	map00400	2.89		
	aroDE; 3-dehydroquinate dehydratase / shikimate dehydrogenase	comp122402_c0_seq2	EC:4.2.1.10 1.1.1.25	map00400	2.48		
	HPD; 4-hydroxyphenylpyruvate dioxygenase	comp121627_c1_seq28	EC:1.13.11.27	map00400		3.45	
	CYP73A; trans-cinnamate 4-monooxygenase	comp110708_c0_seq2	EC:1.14.13.11	map00400		3.15	
Volatiles relative metabolism							
Terpenoid backbone biosynthesis							
	dxs; 1-deoxy-D-xylulose-5-phosphate synthase	comp96886_c1_seq1	EC:2.2.1.7	map00900	2.71	3.18	
	dxs; 1-deoxy-D-xylulose-6-phosphate synthase	comp96886_c2_seq1	EC:2.2.1.7	map00900	3.8	4.49	
	dxs; 1-deoxy-D-xylulose-7-phosphate synthase	comp109831_c0_seq1	EC:2.2.1.7	map00900	4.93	2.39	
	dxs; 1-deoxy-D-xylulose-8-phosphate synthase	comp116264_c0_seq1	EC:2.2.1.7	map00900	2.29	2.42	2.57
	all-trans-nonaprenyl-diphosphate synthase	comp117530_c0_seq2	EC:2.5.1.84 2.5.1.85	map00900	2.14		
	chlP; geranylgeranyl reductase	comp109556_c0_seq1	EC:1.3.1.83	map00900	2.75	2.3	1
	GGPS; geranylgeranyl diphosphate synthase, type II	comp120820_c1_seq1	EC:2.5.1.1 2.5.1.10 2.5.1.29	map00900	4.79	4.58	
	HMGCR; hydroxymethylglutaryl-CoA reductase (NADPH)	comp117873_c3_seq2	EC:1.1.1.34	map00900		2.88	
	ispH; 4-hydroxy-3-methylbut-2-enyl diphosphate reductase	comp113456_c0_seq1	EC:1.17.1.2	map00900		2.05	
	DHDDS; ditrans,polycis-polyprenyl diphosphate synthase	comp109846_c0_seq1	EC:2.5.1.87	map00900		−2.56	−1.14
Monoterpenoid biosynthesis							
	(+)-neomenthol dehydrogenase	comp115928_c0_seq1	EC:1.1.1.208	map15095		3.54	
Diterpenoid biosynthesis							
	gibberellin 3-beta-dioxygenase	comp109499_c0_seq1	EC:1.14.11.15	map00904	3.08	2.62	
	gibberellin 2-oxidase	comp110716_c0_seq2	EC:1.14.11.13	map00904		6.36	
	gibberellin 20-oxidase	comp96433_c1_seq1	EC:1.14.11.12	map00904		2.1	
Tropane, piperidine and pyridine alkaloid biosynthesis							
	EHMT; euchromatic histone-lysine N-methyltransferase	comp119529_c0_seq11	EC:2.1.1.43	map00310	−3.13		
	TR1; Tropinone reductase 1	comp115174_c0_seq6	EC:1.1.1.206	map00310	6.27	6.19	−6.67
	TAT; tyrosine aminotransferase	comp119579_c0_seq3	EC:2.6.1.5	map00310		2.72	
Carotenoid biosynthesis							
	crtB; phytoene synthase	comp118863_c3_seq1	EC:2.5.1.32	map00906		2.76	
	PDS; 15-cis-phytoene desaturase	comp117265_c0_seq1	EC:1.3.5.5	map00906		3.46	
	lcyB; lycopene beta-cyclase	comp109180_c0_seq1	EC:5.5.1.19	map00906		8.16	
	ZEP; zeaxanthin epoxidase	comp119396_c0_seq1	EC:1.14.13.90	map00906		4.15	
	NCED; 9-cis-epoxycarotenoid dioxygenase	comp118160_c0_seq2	EC:1.13.11.51	map00906		6.16	
	crtZ; beta-carotene 3-hydroxylase	comp104888_c0_seq1	EC:1.14.13.129	map00906		2.79	−2.56
	LUT5; beta-ring hydroxylase	comp118802_c0_seq3	EC:1.14.-.-	map00906			−2.5
Phenylpropanoid biosynthesis							
	cinnamyl-alcohol dehydrogenase	comp99291_c1_seq1	EC:1.1.1.195	map00940		8.5	2.82
	beta-glucosidase	comp121918_c0_seq5	E3.2.1.21	map00940		5.01	
	phenylalanine ammonia-lyase	comp115247_c0_seq34	EC:4.3.1.24	map00940		2.14	
	coniferyl-aldehyde dehydrogenase	comp113314_c0_seq1	EC:1.2.1.68	map00940		3.45	
Steroid biosynthesis							
	SQLE; squalene monooxygenase	comp112679_c3_seq1	EC:1.14.13.132	map00100		2.89	
	FDFT1; farnesyl-diphosphate farnesyltransferase	comp113462_c0_seq1	EC:2.5.1.21	map00100		2.84	
	DET2; steroid 5-alpha-reductase	comp82870_c0_seq1	EC:1.3.1.22	map00100			−2.56
Naphthalene degradation							
	frmA; S-(hydroxymethyl)glutathione dehydrogenase / alcohol dehydrogenase	comp110100_c0_seq1	EC:1.1.1.284 1.1.1.1				13.84
The other important metabolism							
Isoquinoline alkaloid biosynthesis							
	TAT; tyrosine aminotransferase	comp119529_c0_seq11	EC:2.1.1.43	map00950	−3.13		
	tyrosine decarboxylase	comp100681_c0_seq1	EC:4.1.1.25	map00950	7.51	4.26	
Benzoate degradation							
	acyP; acylphosphatase	comp107772_c0_seq1	EC:3.6.1.7	map00362		2.26	
	paaH; 3-hydroxybutyryl-CoA dehydrogenase	comp103732_c0_seq1	EC:1.1.1.157	map00362		−2.08	
Butanoate metabolism							
GABA	glutamate decarboxylase	comp118227_c0_seq2	EC:4.1.1.15	map00650	2.2	2.07	−1.27
	POP2; 4-aminobutyrate---pyruvate transaminase	comp112962_c5_seq1	EC:2.6.1.96	map00650	2.78	3.51	
	ECHS1; enoyl-CoA hydratase	comp110838_c0_seq1	EC:4.2.1.17	map00650			29.37
	HADHA; enoyl-CoA hydratase / long-chain 3-hydroxyacyl-CoA dehydrogenase	comp110054_c0_seq1	EC:4.2.1.17 1.1.1.211	map00650			18.24
Flavonoid biosynthesis							
	CYP73A; trans-cinnamate 4-monooxygenase	comp110708_c0_seq2	EC:1.14.13.11	map00941	2.57	3.15	
	caffeoyl-CoA O-methyltransferase	comp98665_c1_seq1	EC:2.1.1.104	map00941	2.04	3.75	
	FLS; flavonol synthase	comp105197_c1_seq1	EC:1.14.11.23	map00941	3.25		
	flavonoid 3'-monooxygenase	comp112481_c0_seq1	EC:1.14.13.21	map00941	2.04		
	ANR; anthocyanidin reductase	comp115221_c0_seq4	EC:1.3.1.77	map00941	2.48		
	flavonoid 3'-monooxygenase	comp112481_c0_seq1	EC:1.14.13.21	map00941		2.39	
	ANR; anthocyanidin reductase	comp115221_c0_seq4	EC:1.3.1.77	map00941		2.96	
	LAR; leucoanthocyanidin reductase	comp112210_c1_seq1	EC:1.17.1.3	map00941		2.31	
	DFR; bifunctional dihydroflavonol 4-reductase/flavanone 4-reductase	comp79789_c0_seq1	EC:1.1.1.219 1.1.1.234	map00941		2.09	
	CHS; chalcone synthase	comp124817_c0_seq1	EC:2.3.1.74	map00941			1.87
Metabolism of xenobiotics by cytochrome P450							
	alcohol dehydrogenase	comp80279_c0_seq1	EC:1.1.1.1	map00001	6.97	5.95	
	GST; glutathione S-transferase	comp104802_c0_seq1	EC:2.5.1.18	map00001		−4.54	1.05
	UGT; glucuronosyltransferase	comp115835_c0_seq1	EC:2.4.1.17	map00001			19.13
	frmA; S-(hydroxymethyl)glutathione dehydrogenase / alcohol dehydrogenase	comp110100_c0_seq1	EC:1.1.1.284 1.1.1.1	map00001			13.84

### JA responsive pathways in MeJA-induced tea leaves

**Fig. 3:**
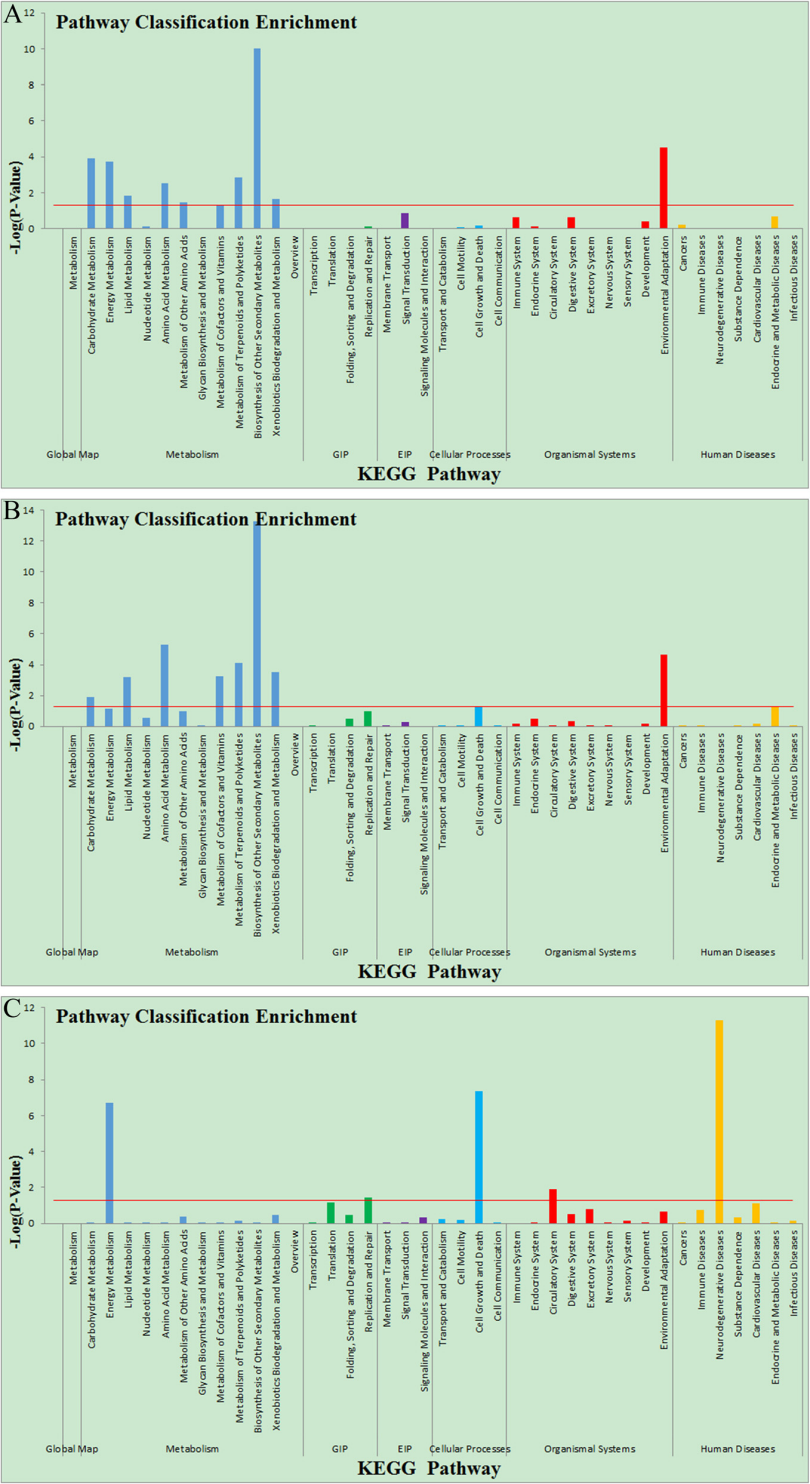
KEGG enrichment assigned to tea unigenes. **a**. KEGG enrichmen of 12 h methyl jasmonate-induced tea leaves; **b**. KEGG enrichmen of 24 h methyl jasmonate-induced tea leaves; **c**. KEGG enrichmen of 48 h methyl jasmonate-induced tea leaves. Note: red line represents the *p* value = 0.05

Interestingly, six DEGs were closely associated with the α-linolenic acid metabolism that finally leads to JA biosynthesis (Table 3; Fig. 3). The JA signaling pathway is the most important signal-transduction pathway in response to predation and pathogen attack, acting as a “master switch” [7, 29–31]. It may play a central role to trigger expression of those DEGs encoding lipoxygenase (EC:1.13.11.12), acetyl-CoA acyltransferase 1 (EC: 2.3.1.16), two kinds of oxidase (EC:1.3.3.6; EC:5.3.99.6) and jasmonate O-methyltransferase (EC:2.1.1.141). Previous reports suggested that genes could perceive and respond to local and systemic signals generated by external stimuli, including MeJA itself [32–36]. During exogenous MeJA treatment, the expression level of jasmonate O-methyltransferase, which catalyzes directly the substrates of (−)-JA biosynthesis, was upregulated by 7.52-fold compared with the control (Additional file 2: Figure S1, Additional file 5: Figure S3; Table 3). Free-acid JA might not be able to move across the cellular membrane without a carrier because of its acidic nature; nonetheless, MeJA could diffuse to distal parts of plant via the vapor phase or by intercellular migration [37, 38]. It is possible for exogenous MeJA to transfer into tea leaves, where it triggers a series of fatty acid pathways resulting in biosynthesis of more JA and JA-conjuncts. Finally, the JA-conjuncts may trig the whole plant’s JA pathway [39–42].

### Responses of biosynthetic pathways of the flavor volatile compounds to MeJA

#### MeJA affects Fatty acid metabolism pathways

Jasmonic acid and its volatile methyl ester act as phytohormones, and are involved in plant responses to stress and developmental processes. During MeJA treatment, the fatty acid pathways are the first to respond, producing low molecular volatiles. At least 13 enzymes are involved in the biosynthetic pathway leading to volatiles formation, including lipoxygenase (LOX) (EC:1.13.11.58), acetyl-CoA acyltransferase 1 (ACAA1) (EC:2.3.1.16), allene oxide cyclase (EC:5.3.99.6) and acyl-CoA oxidase (EC:1.3.3.6) (Additional file 2: Figure S1).

In plants, fatty acids are stored as triacylglycerides; therefore, enzymatic oxidative degradation of lipids is preceded by the action of acyl hydrolase, liberating the free fatty acids from acylglycerols. Saturated and unsaturated volatile C_6_ and C_9_ aldehydes and alcohols are important contributors to the characteristic aromas of tea, which are described as a “fresh green” odor. The short-chain aldehydes and alcohols are mostly produced by plants in response to external stress and play an important role in the plants defense strategies (Additional file 3: Figure S2A) [43–47]. Quantitatively and qualitatively, the majority of plant volatiles originate from saturated and unsaturated fatty acids. In tea plants, we identified many fatty acid-derived straight-chain alcohols, aldehydes, ketones, acids, esters and lactones, which are formed by three basic processes: α -oxidation, β-oxidation and the lipoxygenation. According to Table 1, C_6_-C_9_ volatiles: 2-ethoxy-Butane, 1-ethoxy-Butane, Cyclohexane, 1-ethoxy-Pentane, 2-methyl-Decane, and 2,2-dimethyl-Propanal, increased immediately in MeJA-induced tea leaves. In addition, large amounts of volatiles such as: 2-ethyl-1-Hexanol, 2-methyl-Decane, Acetaldehyde, 2,4-dimethyl-Heptane, 4-methyl-Octane, 1-methoxy-3-methyl-Butane, were synthesized compared with MeJA-untreated tea leaves. The results shown in row d of Table 1 suggested that these volatiles were released quickly into the external environment in response to recognition of exogenous threat. In particular, 2-Hexenal is a representative volatile compound synthesized by fatty acid pathways, compared with the control, after 12 h of MeJA treatment the 2-Hexenal content had increased massively; subsequently, it recovered to a normal level after 24 h of treatment. Interestingly, during the procedure, 2-Hexenal was released quickly from tea leaves, suggesting that it had an important impact on abiotic stress.

Taking these results together, in tea leaves subjected to the abiotic stress if MeJA treatment, the JA pathway stimulation upregulates the fatty acid pathways, resulting in rapid changes to the C_6_–C_9_ volatiles profile.

#### MeJA affects terpenoids biosynthesis pathways

**Fig. 4:**
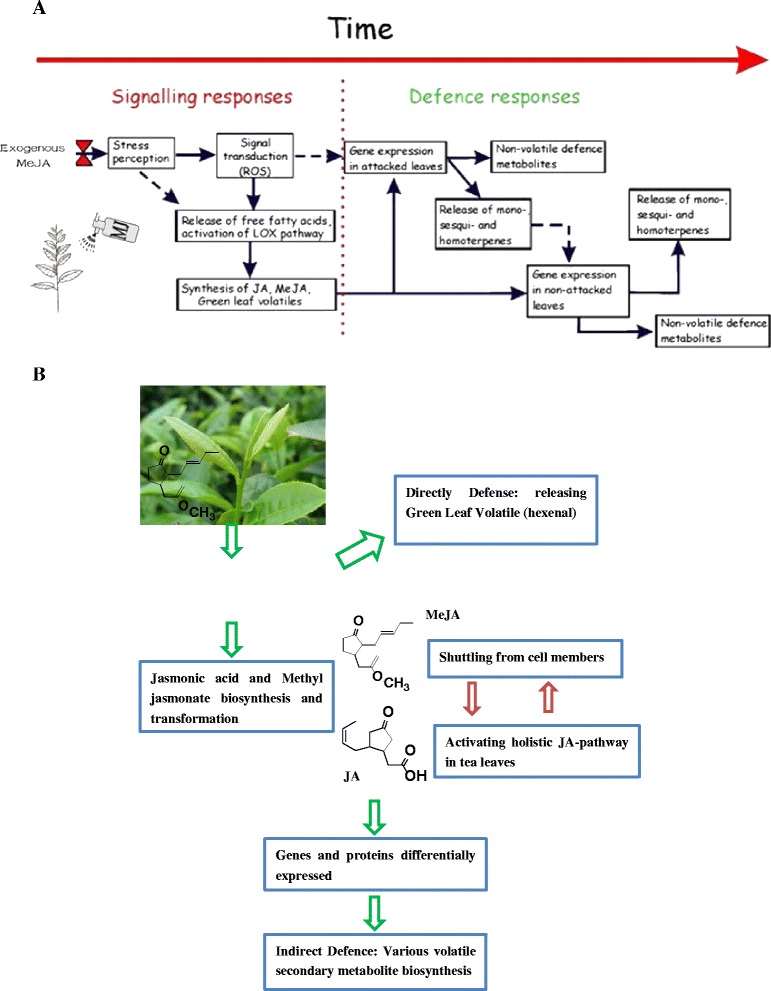
Biology response to of time-dependent methyl jasmonate treatment in tea leaves. **a**. exogenous methyl jasmonate could lead to a rapid, within minutes, oxidative burst and release of free fatty acids and further cascade of events includes activation of defense gene expression that leads to synthesis of a variety of volatile isoprenoids and also production of non-volatile defense compounds such as polyphenols. **b**. The octadecanoid signaling pathway for some gene expression in tea leaves: Exogenous MeJA could in a great degree lead to the activation of lipoxygenase pathway that results in release of green leaf volatiles (a variety of C_6_ aldehydes) and synthesis of jasmonate and methyl jasmonate which could further elicit the JA pathway in the whole tea plant

The most diverse family of natural products is the terpenoids, with over 40,000 different structures. Various plants produce terpenoids, including volatile ones and non-volatile ones. The volatile terpenoids (hemiterpenoids[C_5_], monoterpenoids[C_10_], sesquiterpenoids[C_15_] and some diterpenoids[C_20_]) are important in interactions between plants and insect herbivores, and are implicated in exogenous elicitor-induced general defense or stress responses (Figs. 2a and 4) [48–52]. Despite their diversity, all terpenoids are derived from the common building unit isopentenyl diphosphate (IDP) and its isomer, dimethylallyl diphosphate (DMADP). Generally speaking, the two 5C building blocks (DMADP and IDP) are formed via two independent pathways: the mevalonic acid (MEV) pathway and the 2C-methyl-D-erythritol-4-phosphate (MEP) pathway. IDP and DMADP derived from the cytosolic MEV pathway could serve as precursors for the biosynthesis of the sesquiterpenes (C_15_) and triterpenes (C_30_), whereas the plastidial MEP pathway provides precursors for the biosynthesis of the monoterpenes (C_10_), diterpenes (C_20_), and tetraterpenes (C_40_) [53–55].

According to the results of RNA-Seq, the expressions of 10 DEGs related to the terpenoids backbone biosynthesis pathway were upregulated by treatment of MeJA (Table 3). The content isopentenyl diphosphate should be promoted by the higher expression level of Hydroxymethylglutaryl-CoA reductase (HMG-CoA) (EC:1.1.1.34) mRNA, which was increased by 2.88-fold after MeJA_24h treatment. The increased expression of ispH (EC:1.17.1.2) mRNA could increase the biosynthesis of IDP and DMADP. The high expression of GGPS (EC:2.5.1.1 2.5.1.10 2.5.1.29) mRNA, which was increased by 4.79-fold after MeJA treatment, could also promote the synthesis of GDP, GGDP, and FDP (Additional file 6: Figure S4, Additional file 7: Figure S6).

According to our metabolite results, we found that the levels of the above-mentioned flavor aroma compounds were higher in treated compared with untreated samples (Table 1). In particular, Linalool and Geraniol, which contribute significantly to tea aroma quality with a floral smell, increased by 1.91 and 2.63 μg/2 g, respectively.

The accumulation of GDP, GGDP, and FDP, could promote the production of terpenoids biosynthesis (C_10_–C_40_). Note that the expression level of terpene synthase (TPS), which is an important hydrolyzing enzyme for releasing tea aroma, showed no significant difference in expression between MeJA-treated tea leaves the controls. Linalool and Geraniol are synthesized from the precursors GDP, GGDP, and FDP; therefore, speculated that the contents of these precursors were the limiting factors for aroma volatiles release from tea leaves. The MeJA treatment significantly increased terpenoids biosynthesis by upregulating the expressions of genes related to the terpenoids backbone biosynthesis pathway.

#### MeJA affects phenylpropanoids and some amino acid-derived volatiles biosynthesis pathways

Aldehydes and alcohols derived from the degradation of branched-chain and aromatic amino acids constitute a class of highly abundant volatiles in tea; however, their metabolic pathways have been barely analyzed. The catabolism of amino acids has been analyzed in detail, and is initiated by amino transferases forming 2-ketoacids that serve as substrates for three biochemical reactions: (i) oxidative decarboxylation to carboxylic acids; (ii) decarboxylation to aldehydes; and (iii) reduction to 2-hydroxyacids. Compounds derived from phenylalanine, such as phenylacetaldehyde and 2-phenylethanol, are abundant in various fruits, such as strawberry, tomato and grape, and in tea [17, 56].

Phenylpropanoids/benzenoids and volatile compounds, primarily derived from phenylalanine, contribute to the aromas and scents of many plant species and play important roles in plant communication with the environment [57, 58]. Treatment by MeJA affected the phenylpropanoids biosynthesis pathway. The expression of phenylalanine ammonia-lyase (EC:4.3.1.24) increased by 2.14-fold, which could lead directly to the production of more Cinnamic acid; the high content of this precursor ensures sufficient substrates to produce benzaldehyde and benzylalcohol. The high expression level of beta-glucosidase in this pathway could lead to a greatly increased content of coumarin (Table 3; Additional file 5: Figure S3, Additional file 8: Figure S5). Moreover, phenylethyl alcohol and methyl salicylate are common components of floral scents in plants [59]. During the first 12 h, these two compounds were massively synthesized, which would affect the quality of tea aroma.

#### MeJA affects Carotenoid-derived volatiles biosynthesis pathways

Carotenoid-derived volatiles also contribute to the aroma and quality of tea. The transcriptome results showed that at least seven DEGs involved in the carotenoid pathway were affected by MeJA treatment. The expressions of crtB, PDS and NCED increased by 2.76-, 3.45- and 6.16-fold, respectively, in 24 h MeJA-treated tea leaves compared with the controls. Increased expression of these three DEGs would result in upregulated biosynthesis of ξ-carotene (Tables 1 and 3).

#### Validation of some important DEGs profiling using RT-qPCR

**Fig. 5:**
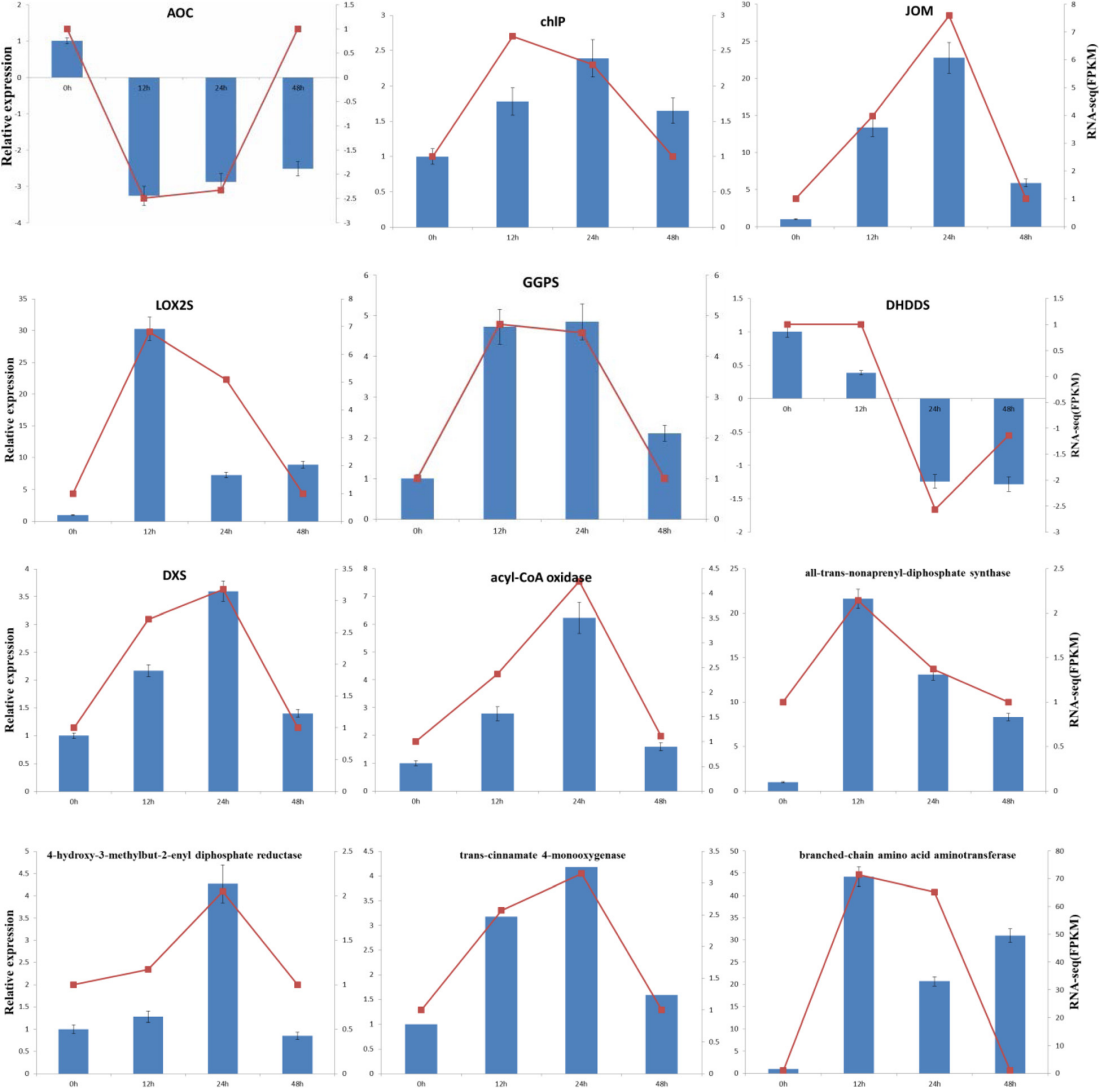
Quantitative RT-qPCR validations. A total of 11 genes were selected for the quantitative RT-qPCR experiments. Of them, AOC(allene oxide cyclase), chlP(geranylgeranyl reductase), JOM(jasmonate O-methyltransferase), LOX2S(lipoxygenase), GGPS(geranylgeranyl diphosphate synthase, type II), DHDDS(ditrans,polycis-polyprenyl diphosphate synthase) and DXS(1-deoxy-D-xylulose-5-phosphate synthase), acyl-CoA oxidase, all-trans-nonaprenyl-diphosphate synthase, 4-hydroxy-3-methylbut-2-enyl diphosphate reductase, trans-cinnamate 4-monooxygenase, and branched-chain amino acid aminotransferase

In order to experimentally validate the reliability of these important differential expressed genes obtained from the assembled transcriptome and profiling of gene expression obtained by RNA-Seq data, a total of 11 key unigenes involved in the biosynthesis of α-linolenic acid degradation (LOX2S, AOC, JOM, acyl-CoA oxidase) and terpenoid backbones biosynthesis (chlP, GGPS, DHDDS, DXS, 4-hydroxy-3-methylbut-2-enyl diphosphate reductase) and some other important pathways (all-trans-nonaprenyl-diphosphate synthase, trans-cinnamate 4-monooxygenase, and branched-chain amino acid aminotransferase) were selected for RT-qPCRs (Fig. 5).

The results suggest that the assembled transcripts are reliable and the designed primer pairs are suitable for the subsequent expression experiments. Based on the delta-delta Ct (2-ΔΔCt) method, relative expression levels of the selected unigenes were calculated and compared among the four different tissues. Mostly, the expression patterns of these genes detected by RT-qPCR were mainly consistent with those from RNA-Seq data. Overall, RT-qPCR experiments confirmed that the unigenes obtained from the assembled transcriptome are trustworthy and gene expression profiles from RNA-Seq data should be believable.

## Conclusions

In the study, we carried out an RNA-Seq analysis of MeJA-elicited transcriptional changes to identify the candidate genes involved in the biosynthesis of secondary metabolites in tea leaves, especially the biosynthesis of volatiles. In total, we obtained 19245, 18614, 11890 DEGs in the MeJA_12h, MeJA_24h and MeJA_48h samples. Most of the DEGs that we picked out in KEEG pathways involved in secondary metabolic pathways, especially, terpenoids and phenylpropanoids pathway, in addition to transcripts associated with MeJA biosynthesis and plant stress responses.

Some of the MeJA upregulated transcripts are potential candidates for regulation of jasmonic acid biosynthesis. Among these, jasmonate O-methyltransferase changed in a great degree to a7.52-fold. Moreover, C-acyltransferases and oxidaseswere also identified. The data also suggest that MeJA responsiveness of the MEP and MEV pathways provide 5C building blocks for the biosynthesis of the diverse terpene metabolites. We found the promoted expression levels of 1-deoxy-D-xylulose-phosphate synthase (DXS), all-trans-nonaprenyl-diphosphate synthase, geranylgeranyl reductase, geranylgeranyl diphosphate synthase (type II), hydroxymethylglutaryl-CoA reductase and 4-hydroxy-3-methylbut-2-enyl diphosphate reductase actually changed the volatile metabolites. However, we did not identify MeJA-induced expression of the terpene synthases, such as: linalool synthase, eugenol synthase, and polyphenol oxidase, and, therefore, could not substantiate earlier reports of MeJA-induced biosynthesis of the corresponding metabolites in tea leaves. However, we did identify, for the first time, MeJA-induced upregulation of genes associated with terpenoid backbone biosynthesis.

The research may lead us a much comprehensive understanding of tea respond to MeJA treatment resulting in volatile compounds changed in tea leaves, these results here also represent the massive genetic resource for tea volatile biosynthesis and will provide a totally new insight into the genomic research in the area.

## Methods

### Plant materials and MeJA treatment

Two-year old Jinxuan, a cultivar of the tea plant (*Camellia sinensis*), was planted in the greenhouse of the Tea Research Institute, Chinese Academy of Agricultural Sciences. Samples were treated and prepared in Spring. All the experiments were carried out in triplicate, separately in March, April, and May. Two thousand individual tea plants were evenly sprayed with 8 L 0.25 % (v/v) water solution of MeJA, which was pre-dissolved in 25 ml ethanol as the treated samples. The fresh tea leaves were plucked after 12 h, 24 h, and 48 h of treatment (one bud with the second leaves). The control plants (CK) were sprayed with 8 L pure water (25 ml ethanol was pre-dissolved) and then processed the same procedure as the MeJA treated samples. The plucked tea leaves were immediately put into liquid nitrogen for subsequent total RNA isolation.

To minimize biological variance, each sample was harvested in three independent biological replicates of equal weight and subsequently pooled for sequencing and volatile analysis. RNA-seq was analyzed twice, MeJA-treated samples were marked as meja_12h-1, meja_24h-1, meja_48h-1; meja_12h-2, meja_24h-2, meja_48h-2; the control samples were marked as ck-1,ck-2 (the control samples were the mixtures of four 0 h, 12 h, 24 h,48 h which picked within the same time as the MeJA-treated tea), and the volatile analysis was performed for three replications.

### GC*GC-TOF/MS analysis

#### Sample preparation

Two grams (fresh matter) of leaf tissues, which were finely powdered in liquid nitrogen and crushed by a Multi-Beads Shocker (2000 rpm, 15 s, Yasui Kikai Corporation, Japan), were extracted with 5mlof diethyl ether containing 42 nmol ethyl n-decanoate as an internal standard at ambient temperature for 17 h in the dark. The extract was filtered through a short plug of anhydrous sodium sulfate. One microliter of the filtrate was subjected to Leco GC*GC-TOF/MS analysis.

#### GC conditions

A LECO Pegasus 4D GC*GC–TOF/MS instrument (LECO Corporation, St. Joseph, MI, USA) equipped with an Agilent 6890 N (Agilent, PaloAlto, CA, USA) was used in analyzing the extracts of these tea samples. The first dimension (1D) column was a DB-5MS column of 30 m × 250 μm × 0.25 μm and the second dimension (2D) was a DB-17HT column of 10 m × 100 μm × 0.10 μm (J&W Scientific, Folsom, CA, USA). The temperatures of the GC inlet and transfer line were set at 280 °C and 270 °C, respectively. The carrier gas was 99.9995 % high purity helium at a constant pressure mode. The pressure at the head of the column was 200kPa. Cryogenic modulation was used with a modulation period of 5.0 s. An Agilent 7683B autosampler was used with an injection volume of 1.0 μl in splitless mode. The oven temperature of the first column was held at 60 °C for 3 min, and then ramped to 280 °C (4 °C/min), and held for 5 min at the last temperature. The oven temperature of the second column was initially held at 70 °C for 3 min, and then followed the same program of the first column. The total analysis time was 40.75 min.

#### MS conditions

The temperature of the ion source was set to 220 °C. The MS range was collected from m/z 50 to 650 at 50 spectra per second. The solvent delay time was 150 s. The detector voltage was 1.67 kV and electron energy was −70 eV. A C10–C20 n-alkanes series was analyzed to determine the retention index in the 1D separation. Preliminary identification of compounds was based on similarity comparison of standard MS in NIST05 (National Institute of Standards and Technology, Gaithersburg, MD, USA).

#### Data analysis

The raw data were pre-processed by LECO ChromaTOFTM workstation (version 4.44). Peaks with signal-to-noise ratios (S/N) larger than 100 were extracted, and the corresponding peak areas were calculated by using an extracted ion chromatogram. The software automatically determined the extracted ion chromatogram of each peak after background correction and deconvolution. Two important parameters, the 1D and 2D peak width, may affect the number of peaks; they were set to 25 s (5 heart-cuts × 5 PM time) and 0.4 s, respectively. The software executed the peak merging with an MS similarity of 65 %, and a minimum required S/N of six for all sub-peaks. This helped to produce a peak table with all slices of one analyte together.

### Total RNA isolation and cDNA library construction

Total RNA of each sample was isolated using an RNAprep Pure Plant Kit (Tiangen bio-tek, China), according to the manufacturer’s instructions. The quantity and quality of total RNA were evaluated using a Nanodrop ND-1000 (Nanodrop technologies, Wilmington, DE, USA), gel electrophoresis and an Agilent 2100 analyzer. High quality RNA with a 28S:18S ratio greater than 1.5 and absorbance 260/280 ratio between 1.7 and 2.0 was used for library construction and sequencing.

The cDNA libraries were constructed using Illumina’s kit, following the manufacturer’s protocol (TruSeq RNA Sample Preparation Kits v2, Illumina, San Diego, CA, USA). Magnetic beads with poly A oligos attached were used to purify the mRNA from the total RNA. Fragmentation buffer was added to cleave the mRNA into short fragments. Random hexamer primers were used to generate first-strand cDNA from the fragments, which was transformed into double stranded cDNA using RHase H and DNA polymerase I. A paired-end library was constructed from the cDNA synthesized using a Genomic Sample Prep Kit (Illumina). Fragments of the desired length were purified using a QIAquick PCR Extraction Kit (QIAquick PCR Purification Kit (50), Germany), end repaired and linked with sequencing adapters. AMPureXP beads were used to remove unsuitable fragments, and the sequencing library was then constructed using PCR amplification. Pico green staining and fluorospectrophotometry were used to check the library integrity and an Agilent 2100 quantified it. The multiplexed DNA libraries were then mixed in equal volumes at a normalized concentration of 10nM. The library was then sequenced on the Illumina Miseq platform (by the Shanghai Personal Biotechnology Co., Ltd. Shanghai, China).

### Data filtering and de novo assembly

Raw sequencing reads of all the samples were mixed together to perform filtration using a stringent process and subsequent de novo assembly. Contaminating adaptors were removed, and the reads were screened from the 3′ to 5′ to trim bases with a quality score (Q) <20 using 5 bp windows; reads with a final length less than 50 bp were removed. All the bases in these sequences were defined. De novo transcriptome assembling was carried out step by step as Trinity software performed (http://trinityrnaseq.github.io/). Briefly, the process works with three main steps like so: Firstly, we called it Inchworm which assembles the RNA-seq data into the unique sequences of transcripts, often generating full-length transcripts for a dominant isoform, but then reports just the unique portions of alternatively spliced transcripts. Secondly, Chrysalis clusters the Inchworm contigs into clusters and constructs complete de Bruijn graphs for each cluster. Each cluster represents the full transcriptional complexity for a given gene (or sets of genes that share sequences in common). Chrysalis then partitions the full read set among these disjoint graphs. And finally, Butterfly that processes the individual graphs in parallel, tracing the paths that reads and pairs of reads take within the graph, ultimately reporting full-length transcripts for alternatively spliced isoforms, and teasing apart transcripts that corresponds to paralogous genes.

High quality reads of each sample were remapped to transcripts to estimate the abundance of transcripts. Those transcripts with no reads mapped in all samples were considered errors and removed. All the transcripts were searched against the *Arabidopsis* database, and those with no hits were then BLAST searched against the NCBI non-redundant (nr) database with a cut-off E-value of <1E-5. The top-hit transcripts were selected as unigenes. For the unigenes that did not align to any entries in the databases, the software GetORF was used to predict their open reading frames (ORFs) and ascertain their sequence directions, with default settings except for the parameter “–find” being set 1.

### Gene annotation and comparative expression analysis

Unique sequences were BLAST searched and annotation against the NCBI non-redundant (nr) databases, cluster of orthologous groups of protein (COG) database, Kyoto Encyclopedia of Genes and Genomes (KEGG) database, and gene ontology (GO) database, with a cut-off E-value of 1E-5,. Functional annotations were implied by sequence similarity against the nr database and the annotations of first sequence with highest sequence similarity and clear functional annotation were associated with the corresponding unique sequences. Functional annotation by GO was analyzed against the GO database, and the pathways annotations were retrieved using the internal KEGG information of hits in the GO database.

Genes involved in biosynthesis of the main flavor volatiles were manually identified by BLAST searching. The queries were all from closely-related species, if available, and the genes from *Arabidopsis* thaliana (TAIR, www.arabidopsis.org) were used if they were unknown in *Camellia sinensis*. All the hits with E-value less than 1E-5 in tea leaves were then used as queries to search the GenBank nr database again and were retained if their encoded proteins also were annotated as enzymes involved in volatiles biosynthesis.

### RT-qPCR in validation of candidate genes and levels of gene expression

Elven important unigenes potentially involved in some of the important secondary metabolites biosynthesis pathways were selected for qRT-PCR experiments. Gene-specific primer pairs were designed using Primer primer 5.0 software (Premier Biosoft International), and total RNA was isolated from prepared tea samples using a modified CTAB method, respectively. After treated with DNase I (Tiangen, China), one microgram of RNA was used in reverse transcription with the SuperScript VILO cDNA Synthesis Kit (Invitrogen) according to the manufacturer’s guidelines. The standard curve for each gene was conducted in several dilutions of cDNA, then real-time qPCR was performed using Multicolor Real-Time PCR Detection System (Bio-Rad) with conditions for all reactions were 95 °C for 10 min, 40 cycles of 95 °C for 15 s, followed by 60 °C for 30 s. Melting curve and agarose gel electrophoresis analysis were performed to confirm the PCR specificity. The 18S RNA gene was selected as an internal standard for normalization, and three biological replicates were completed for each gene. The relative expression levels for each unigene were in the different tissues calculated by using the delta-delta Ct (2-ΔΔCt)method. All data were expressed as the mean ± SD after normalization.

### Availability of supporting data

The data set supporting the results of this article is available in the NCBI SRA (Sequence read archive, http://www.ncbi.nlm.nih.gov/sra/) respository under the accession number of SRP060335.

## Additional files

Additional file 1: Table S1. Statistical of the filtered raw date. (DOC 56 kb) Additional file 2: Figure S1. Distribution of contigs, transcripts and unigenes in tea leaves. (DOCX 83 kb) Additional file 3: Figure S2. EggNOG classification of the tea leaves transcriptome. (DOCX 138 kb) Additional file 4: Table S2. Pathway classification of tea leaves. (DOC 58 kb) Additional file 5: Figure S3. Unigenes upregulated by MeJA treatment in α-Linolenic acid metabolism. (DOCX 34 kb) Additional file 6: Figure S4. Unigenes upregulated by MeJA treatment in Terpenoid backbone biosynthesis metabolism. (DOCX 52 kb) Additional file 7: Figure S6. Simplified scheme of the interactions among the biosynthetic pathways responsible for volatiles and non-volatiles stress metabolites in plant. (DOCX 262 kb) Additional file 8: Figure S5. Unigenes upregulated by MeJA treatment in phenylpropanoid biosynthesis metabolism. (DOCX 52 kb)

## Abbreviations

MeJA: Methyl jasmonate
MEP/DOXP: Non-Mevalonate(2C-methyl-D-erythritol-4-phosphate) pathway
DXS: 1-deoxy-D-xylulose-phosphate synthase
GC*GC-TOF/MS: Two-dimensional gas-chromatograph mass-spectrometry
VOCs: Volatile organic components
JA: Jasmonic acid
LOX: Lipoxygenase
ACAA1: Acetyl-CoA acyltransferase 1
IDP: Isopentenyl diphosphate
DMAPP: Dimethylallyl diphosphate
MEV: Mevalonic acid pathway
HMG-CoA: Hydroxymethylglutaryl-CoA reductase
GDP: Geranyl diphosphate
FDP: Farnesyl pyrophosphate
GGPP: Geranylgeranyl pyrophosphate
GGPS: Geranylgeranyl pyrophosphate synthase
