# Erratum to: transcriptional responses and flavor volatiles biosynthesis in methyl jasmonate-treated tea leaves

**DOI:** 10.1186/s12870-017-1076-5

**Published:** 2017-08-09

**Authors:** Jiang Shi, ChengYing Ma, DanDan Qi, HaiPeng Lv, Ting Yang, QunHua Peng, ZongMao Chen, Zhi Lin

**Affiliations:** 1 Key Laboratory of Tea Biology and Resource Utilization of Ministry of Agriculture, Tea Research Institute, Chinese Academy of Agricultural Sciences, 9th South Meiling Road, Hangzhou Zhejiang, 310008 People’s Republic of China; 2 Graduate School of Chinese Academy of Agricultural Sciences, 12 South Street of Zhongguancun, Beijing, 100081 People’s Republic of China

## Erratum

Following publication of this article [1] it has come to our attention that the attribution for the original sources of the images used in two of the figures is missing.

The authors apologise for this omission and would like to make the following corrections to the figure legends for Fig. 4 and Additional file: 7 Figure S6.

Fig. 4 Biology response to of time-dependent methyl jasmonate treatment in tea leaves. (a). exogenous methyl jasmonate could lead to a rapid, within minutes, oxidative burst and release of free fatty acids and further cascade of events includes activation of defense gene expression that leads to synthesis of a variety of volatile isoprenoids and also production of non-volatile defense compounds such as polyphenols. Adapted from Fig. 3 [2] with permission from the authors and with the following modifications: the replacement of an image of a leaf and caterpillar with an image of a plant and spray can

Additional file: 7 Figure S6. Simplified scheme of the interactions among the biosynthetic pathways responsible for volatiles and non-volatiles stress metabolites in plant. Reproduced from Fig. 2 [2] with permission from the authors

The results and conclusions of this article remain unchanged.
