# Supplementary material for: Transcriptional responses and flavor volatiles biosynthesis in methyl jasmonate-treated tea leaves
Source: BMC Plant Biol. 2015 Sep 30;15:233. doi: 10.1186/s12870-015-0609-z (PMC4588909; doi:10.1186/s12870-015-0609-z)
Supplement: Additional file 1: Table S1. — Statistical of the filtered raw date. (DOC 56 kb) [file 12870_2015_609_MOESM1_ESM.doc]

Table S1. statistical of the filtered raw date

| **Sample** | **Reads** | **Clean Reads** | **Clean Data(bp)** | **Useful Reads %** | **Useful Data %** |
| --- | --- | --- | --- | --- | --- |
| 12meja1 | R1 | 22,118,827 | 2,163,522,604 | 60.52% | 59.13% |
|  | R2 | 22,118,827 | 2,158,518,042 |  |  |
|  | Paired | 22,118,827 | 4,322,040,646 |  |  |
| 12meja2 | R1 | 23,146,674 | 2,240,764,926 | 74.36% | 71.94% |
|  | R2 | 23,146,674 | 2,238,109,004 |  |  |
|  | Paired | 23,146,674 | 4,478,873,930 |  |  |
| 24meja1 | R1 | 22,511,812 | 2,180,516,631 | 74.62% | 72.23% |
|  | R2 | 22,511,812 | 2,178,098,781 |  |  |
|  | Paired | 22,511,812 | 4,358,615,412 |  |  |
| 24meja2 | R1 | 21,287,071 | 2,080,941,504 | 60.38% | 58.96% |
|  | R2 | 21,287,071 | 2,076,454,956 |  |  |
|  | Paired | 21,287,071 | 4,157,396,460 |  |  |
| 48meja1 | R1 | 22,703,378 | 2,210,424,899 | 68.03% | 66.05% |
|  | R2 | 22,703,378 | 2,198,258,533 |  |  |
|  | Paired | 22,703,378 | 4,408,683,432 |  |  |
| 48meja2 | R1 | 24,739,529 | 2,408,245,295 | 69.49% | 67.47% |
|  | R2 | 24,739,529 | 2,395,845,339 |  |  |
|  | Paired | 24,739,529 | 4,804,090,634 |  |  |
| ck1 | R1 | 23,949,947 | 2,333,150,950 | 65.93% | 64.19% |
|  | R2 | 23,949,947 | 2,330,799,200 |  |  |
|  | Paired | 23,949,947 | 4,663,950,150 |  |  |
| ck2 | R1 | 23,278,628 | 2,264,370,251 | 65.56% | 63.75% |
|  | R2 | 23,278,628 | 2,263,044,804 |  |  |
|  | Paired | 23,278,628 | 4,527,415,055 |  |  |
