# Supplementary figures and images for: Transcriptional responses and flavor volatiles biosynthesis in methyl jasmonate-treated tea leaves
Source: BMC Plant Biol. 2015 Sep 30;15:233. doi: 10.1186/s12870-015-0609-z (PMC4588909; doi:10.1186/s12870-015-0609-z)

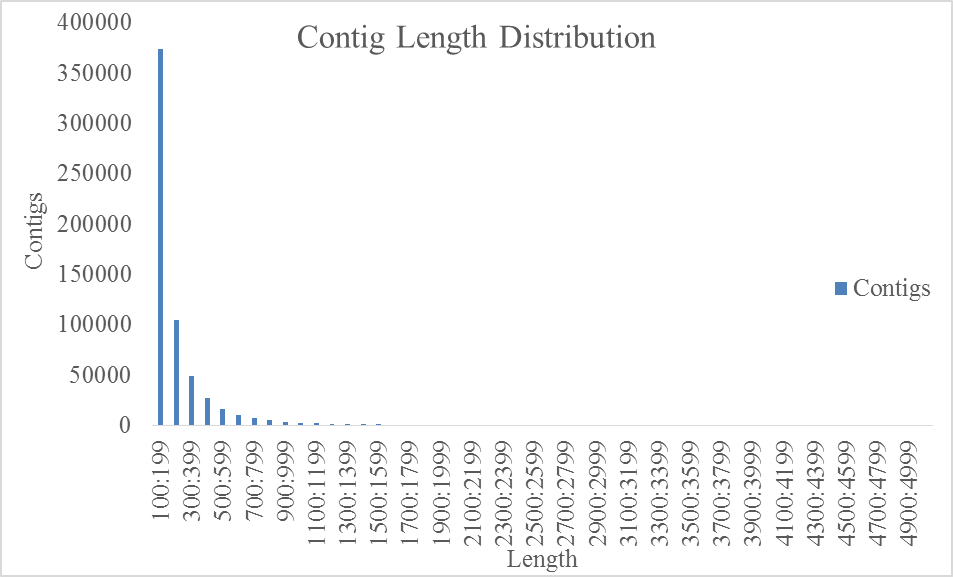


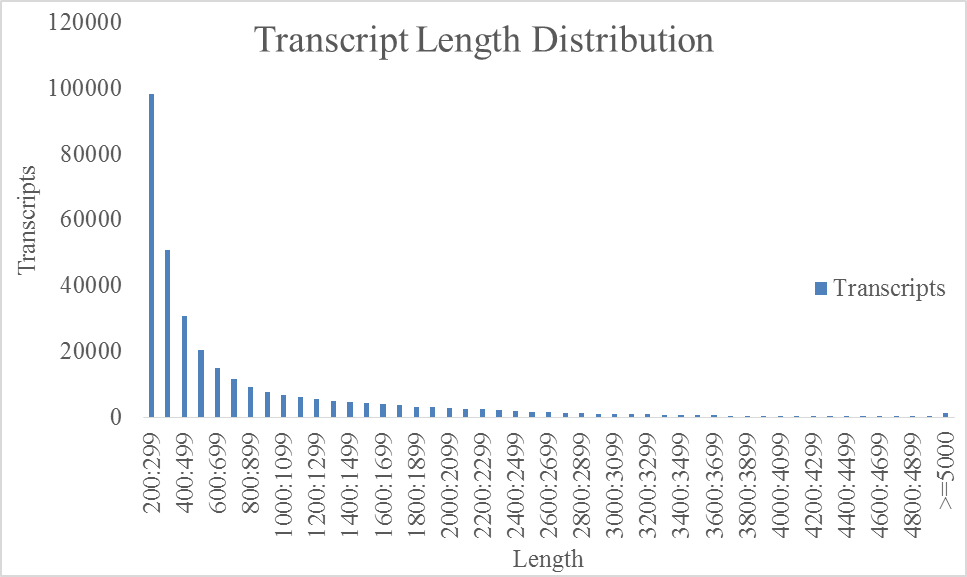


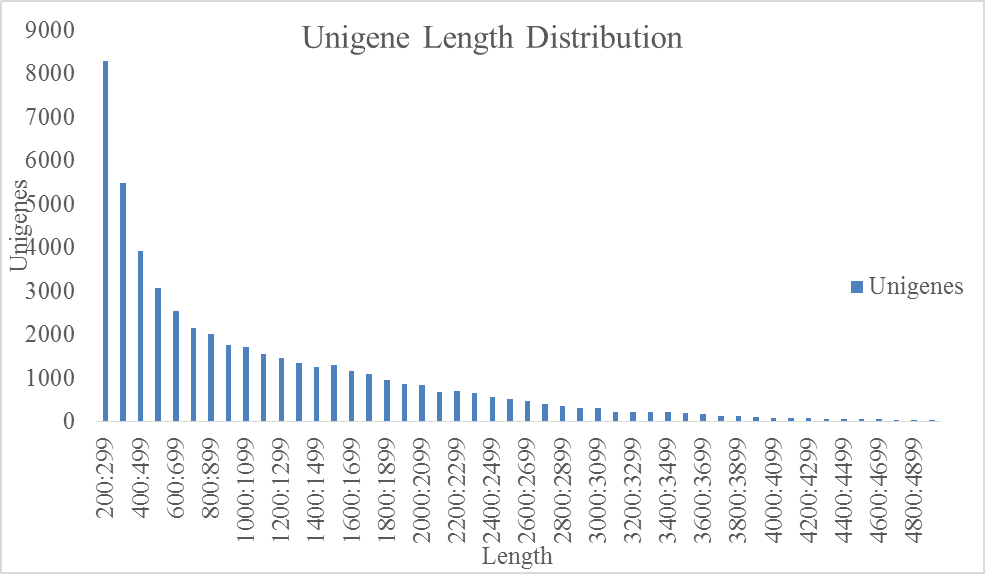

Supplement: Additional file 2: Figure S1. — Distribution of contigs, transcripts and unigenes in tea leaves. (DOCX 83 kb) [file 12870_2015_609_MOESM2_ESM.docx]

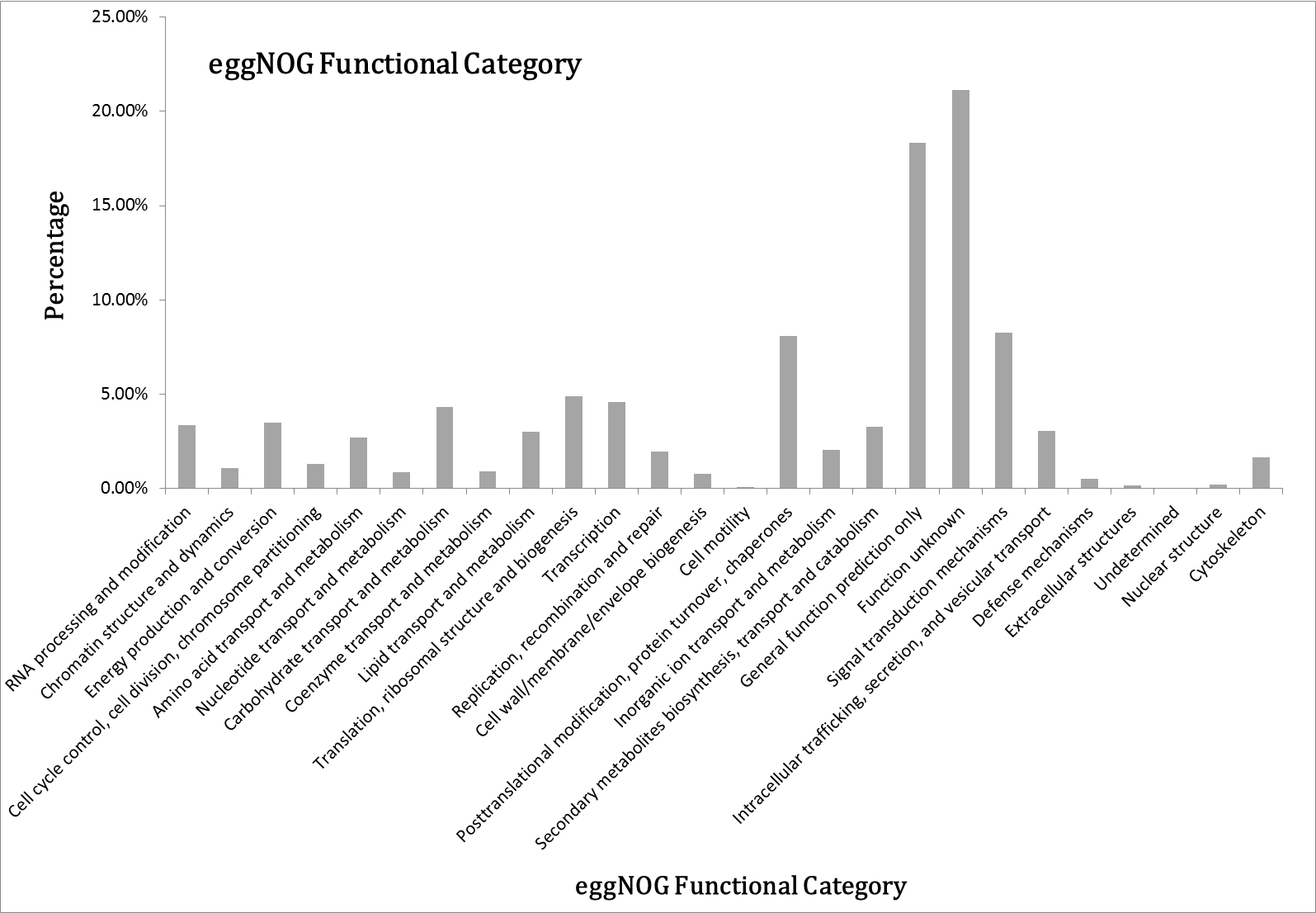

Supplement: Additional file 3: Figure S2. — EggNOG classification of the tea leaves transcriptome. (DOCX 138 kb) [file 12870_2015_609_MOESM3_ESM.docx]

**
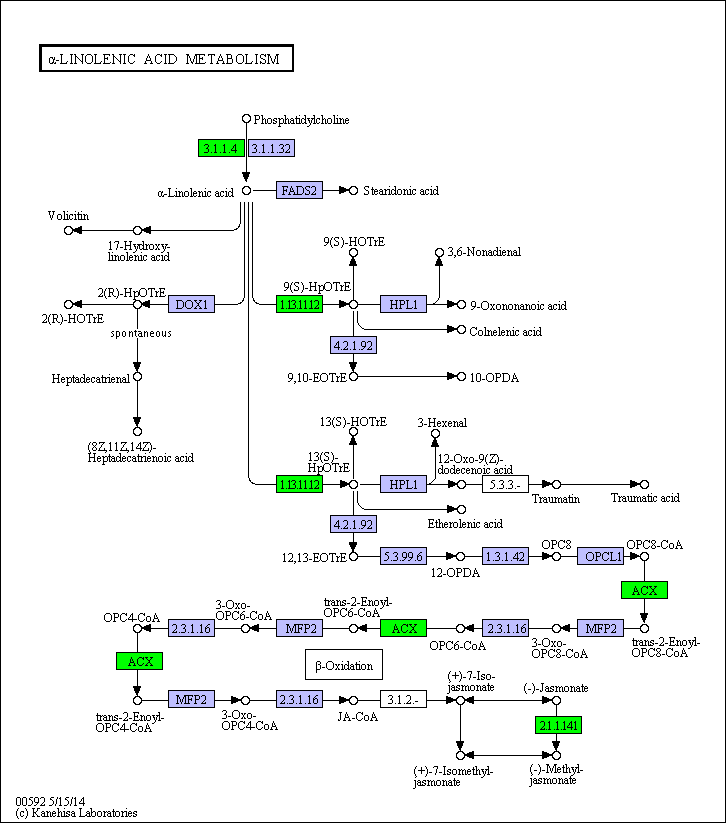
**

Supplement: Additional file 5: Figure S3. — Unigenes upregulated by MeJA treatment in α-Linolenic acid metabolism. (DOCX 34 kb) [file 12870_2015_609_MOESM5_ESM.docx]

**
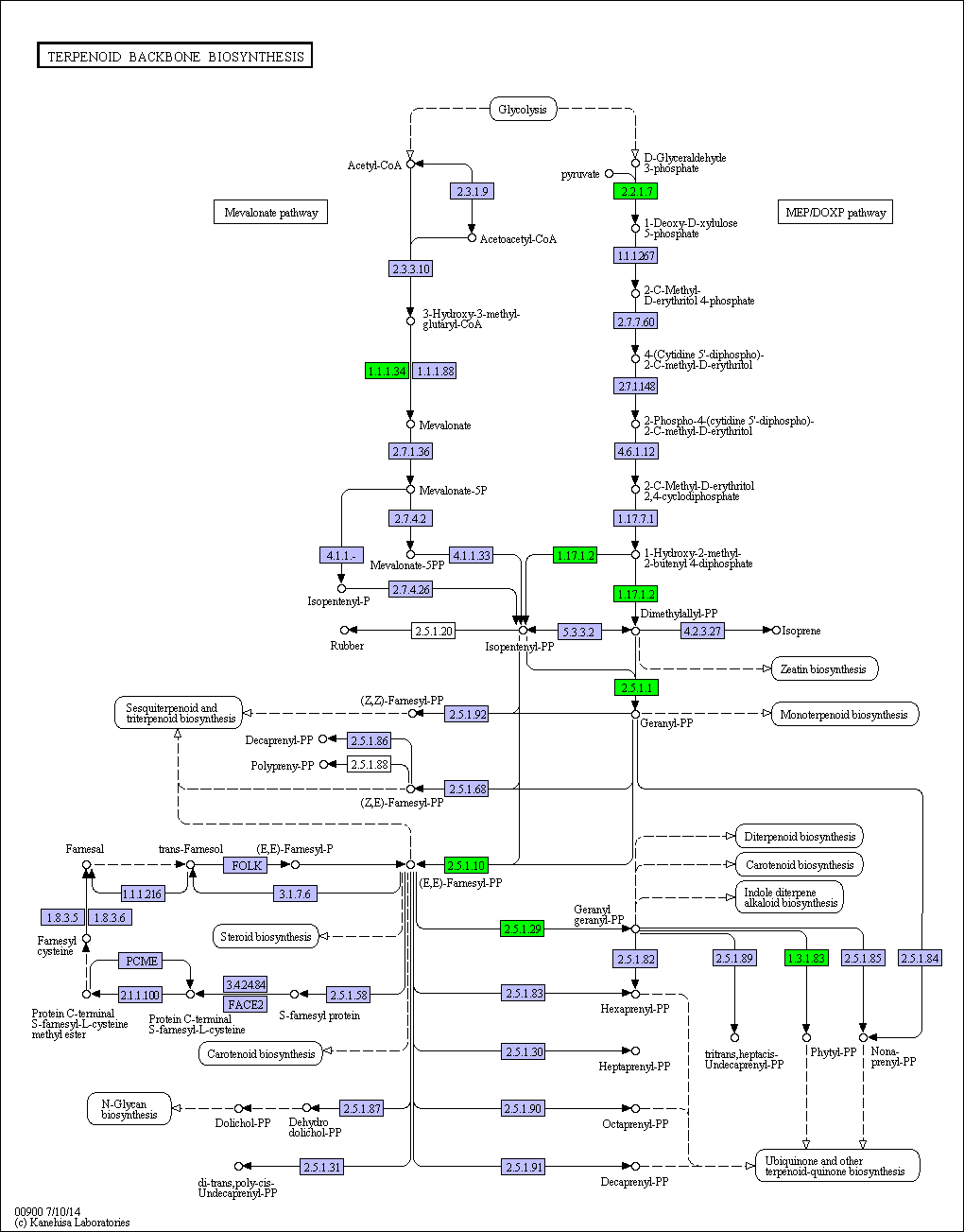
**

Supplement: Additional file 6: Figure S4. — Unigenes upregulated by MeJA treatment in Terpenoid backbone biosynthesis metabolism. (DOCX 52 kb) [file 12870_2015_609_MOESM6_ESM.docx]

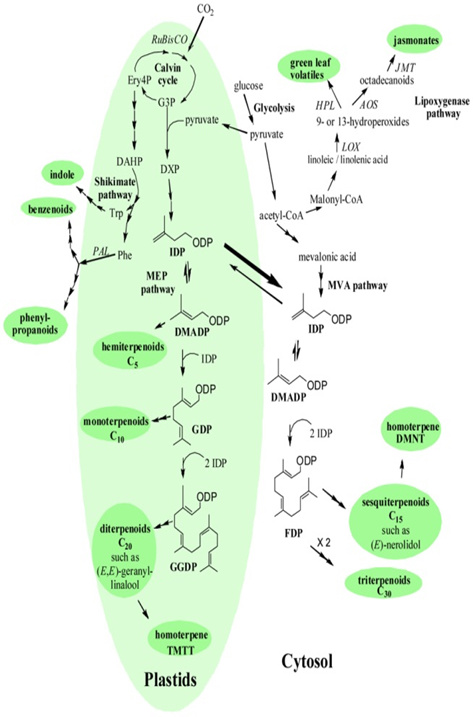

Supplement: Additional file 7: Figure S6. — Simplified scheme of the interactions among the biosynthetic pathways responsible for volatiles and non-volatiles stress metabolites in plant. (DOCX 262 kb) [file 12870_2015_609_MOESM7_ESM.docx]

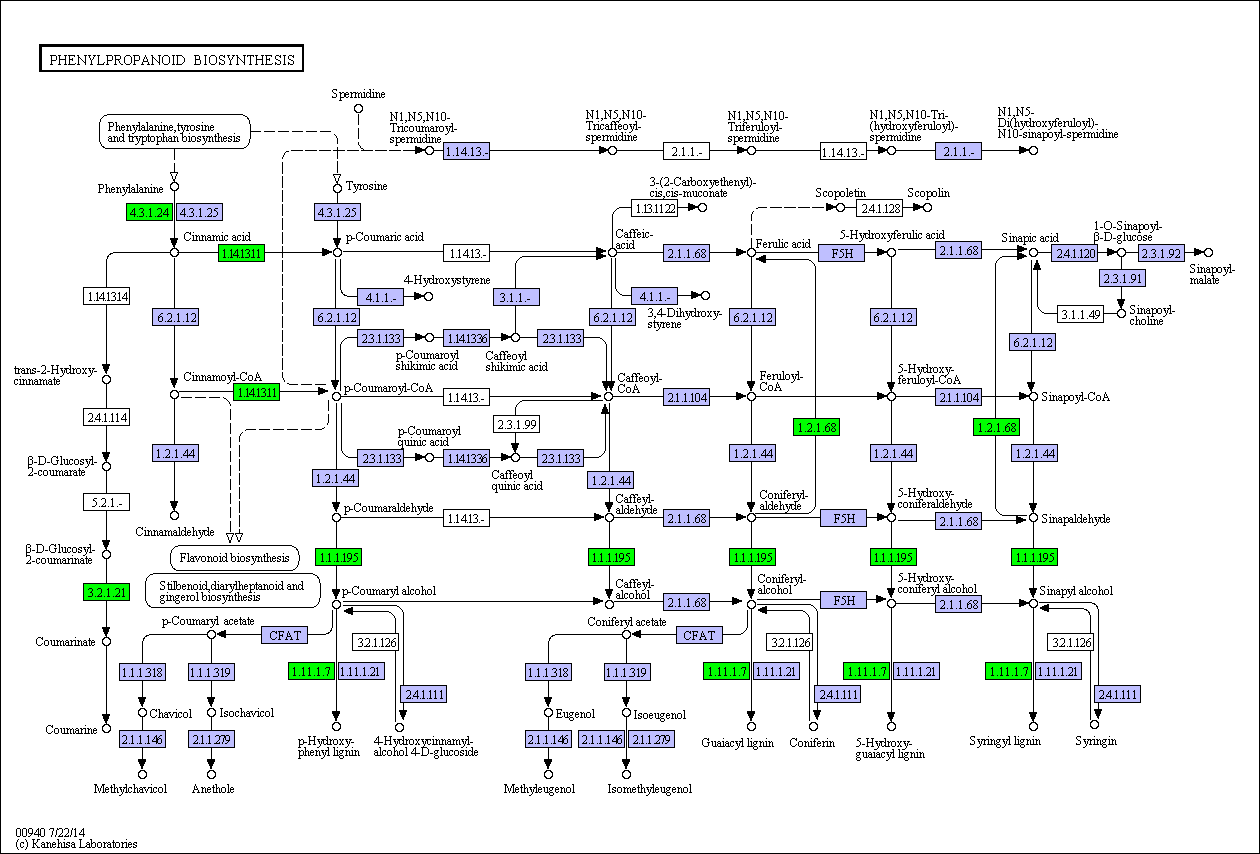

Supplement: Additional file 8: Figure S5. — Unigenes upregulated by MeJA treatment in phenylpropanoid biosynthesis metabolism. (DOCX 52 kb) [file 12870_2015_609_MOESM8_ESM.docx]
