# Supplementary material for: Transcriptional responses and flavor volatiles biosynthesis in methyl jasmonate-treated tea leaves
Source: BMC Plant Biol. 2015 Sep 30;15:233. doi: 10.1186/s12870-015-0609-z (PMC4588909; doi:10.1186/s12870-015-0609-z)
Supplement: Additional file 4: Table S2. — Pathway classification of tea leaves. (DOC 58 kb) [file 12870_2015_609_MOESM4_ESM.doc]

Table S2. Pathway classification of tea leaves

| **Category** | **Pathway** | **Count** |
| --- | --- | --- |
| Metabolism | Carbohydrate Metabolism | 1847 |
|  | Energy Metabolism | 1090 |
|  | Lipid Metabolism | 904 |
|  | Nucleotide Metabolism | 518 |
|  | Amino Acid Metabolism | 1091 |
|  | Metabolism of Other Amino Acids | 375 |
|  | Glycan Biosynthesis and Metabolism | 333 |
|  | Metabolism of Cofactors and Vitamins | 345 |
|  | Metabolism of Terpenoids and Polyketides | 325 |
|  | Biosynthesis of Other Secondary Metabolites | 290 |
|  | Xenobiotics Biodegradation and Metabolism | 364 |
| Genetic Information Processing | Transcription | 448 |
|  | Translation | 626 |
|  | Folding, Sorting and Degradation | 1247 |
|  | Replication and Repair | 510 |
| Environmental Information Processing | Membrane Transport | 64 |
|  | Signal Transduction | 989 |
|  | Signaling Molecules and Interaction | 32 |
| Cellular Processes | Transport and Catabolism | 769 |
|  | Cell Motility | 139 |
|  | Cell Growth and Death | 778 |
|  | Cell Communication | 294 |
| Organismal Systems | Immune System | 607 |
|  | Endocrine System | 465 |
|  | Circulatory System | 138 |
|  | Digestive System | 212 |
|  | Excretory System | 212 |
|  | Nervous System | 567 |
|  | Sensory System | 15 |
|  | Development | 15 |
|  | Environmental Adaptation | 256 |
| Human Diseases | Cancers | 1022 |
|  | Immune Diseases | 168 |
|  | Neurodegenerative Diseases | 1136 |
|  | Substance Dependence | 167 |
|  | Cardiovascular Diseases | 87 |
|  | Endocrine and Metabolic Diseases | 74 |
|  | Infectious Diseases | 1885 |
| Drug Development | Chronology: Antiinfectives | 0 |
|  | Chronology: Antineoplastics | 0 |
|  | Chronology: Nervous System Agents | 0 |
|  | Chronology: Other Drugs | 0 |
|  | Target Based Classification: G Protein-Coupled Receptors | 0 |
|  | Target Based Classification: Nuclear Receptors | 0 |
|  | Target Based Classification: Ion Channels | 0 |
|  | Target Based Classification: Transporters | 0 |
|  | Target Based Classification: Enzymes | 0 |
|  | Structure Based Classification | 0 |
